# Fast, Flexible Closed-Loop Feedback: Tracking Movement in “Real-Millisecond-Time”

**DOI:** 10.1523/ENEURO.0147-19.2019

**Published:** 2019-10-30

**Authors:** Keisuke Sehara, Viktor Bahr, Ben Mitchinson, Martin J. Pearson, Matthew E. Larkum, Robert N. S. Sachdev

**Affiliations:** 1 Institute of Biology, Humboldt University of Berlin, D-10117 Berlin, Germany; 2 Eridian Systems, D-10179 Berlin, Germany; 3 Department of Computer Science, University of Sheffield, Sheffield, S10 2TP United Kingdom; 4 Bristol Robotics Laboratory, University of Bristol and University of the West of England, Bristol, BS16 1QY United Kingdom

**Keywords:** feedback, kinematics, motor, neuro-morphic, somatosensory, virtual reality

## Abstract

One of the principal functions of the brain is to control movement and rapidly adapt behavior to a changing external environment. Over the last decades our ability to monitor activity in the brain, manipulate it while also manipulating the environment the animal moves through, has been tackled with increasing sophistication. However, our ability to track the movement of the animal in real time has not kept pace. Here, we use a dynamic vision sensor (DVS) based event-driven neuromorphic camera system to implement real-time, low-latency tracking of a single whisker that mice can move at ∼25 Hz. The customized DVS system described here converts whisker motion into a series of events that can be used to estimate the position of the whisker and to trigger a position-based output interactively within 2 ms. This neuromorphic chip-based closed-loop system provides feedback rapidly and flexibly. With this system, it becomes possible to use the movement of whiskers or in principal, movement of any part of the body to reward, punish, in a rapidly reconfigurable way. These methods can be used to manipulate behavior, and the neural circuits that help animals adapt to changing values of a sequence of motor actions.

## Significance Statement

Here, we implemented a method for tracking and reacting to movement in real time at low latency, i.e., in 2 ms. We use a neuromorphic camera chip to track movement of a whisker and generate an output based on whisker position. With training, mice learn to move whiskers to virtual target locations. Combined with the recent sophisticated techniques for monitoring and manipulating brain activity, methods like ours can be used to manipulate behavior or neural circuits that help animals adapt to changing values of a sequence of motor actions.

## Introduction

Context matters. Whether a stimulus is large or small, whether it includes the center and surround, whether it is passively applied or actively perceived, whether cortical circuits are active or inactive when the stimulus occurs, all matter for perception of a stimulus. One state of the art development in which context can be changed rapidly, flexibly and in many dimensions at one time is to immerse animals in virtual worlds and to artificially modify the interaction between the animal and the world. In virtual reality systems, animals are placed on floating-balls, treadmills or floating “air-track” systems, and the movement of the platform is tracked in relation to a streaming visual or “real” world presented to the animal (Hölscher et al., 2005; Dombeck et al., 2007; Harvey et al., 2009; Sofroniew et al., 2014; Nashaat et al., 2017; Voigts and Harnett, 2018; Dominiak et al., 2019). These systems can even be used to introduce counterfactual features into the “real” sensory environment, creating illusions that can be used to understand how the brain interprets the external world, creates representations of the world, or remembers features of the world (Keller et al., 2012). Combined with recent genetic, optical or electrophysiological techniques, these virtual-reality systems provide amazing opportunities for studying learning, memory and perception.

While these systems are extraordinarily powerful, they are primarily based on real-time tracking of artificial elements, i.e., the movement of a ball, platform, or a treadmill, and not on tracking the aspect of behavior that the brain directly controls, the body. The major challenge for generating feedback in real time is that for feedback to be meaningful it has to be fast; consequently, when behavioral events occur, they have to be tracked accurately and rapidly. Although it is becoming obvious that behaviors occur in multiple sensorimotor dimensions simultaneously (Musall et al., 2018; Dominiak et al., 2019; Stringer et al., 2019), the dominant approach in systems neuroscience is still to track behavior in a single dimension: a cue triggers a movement and the endpoint of movement is fixed inflexibly at a single spatial location detected by a contact, or a beam break (Evarts, 1966, 1968; Bermejo et al., 1996; Bermejo et al., 1998; Sachdev et al., 2001; O’Connor et al., 2010; Stüttgen and Schwarz, 2010). A powerful and promising new avenue, that has the potential to be more flexible, has been to use image-processing methods that, in combination with machine vision approaches, extract a certain feature of the animal body from video frames (Mathis et al., 2018). However, currently these approaches suffer from a high order of data parallelism and redundancy at each step of data processing, i.e., data acquisition, data transfer to the host computer, and conversion of the acquired frames. Consequently, these systems work at latencies of 50–100 ms, which make them unsuitable for tracking fast events such as whisker moving at frequencies up to 25 Hz (Forys et al., 2018; Štih et al., 2019).

Another solution has been to perform parallel processing at the level of sensors, thus reducing the computation time and permitting real-time tracking. The Pixy camera is one iteration of this solution; it preprocesses objects based on a color code (Nashaat et al., 2017). The dynamic vision sensor (DVS) is another realization of a sensor-level computation approach (Delbruck, 2008). It is a retina-inspired neuromorphic camera where each sensor unit of the camera can be in principle viewed as a difference filter that detects a change in luminance at given position. Compared to the Pixy camera, it has a transfer speed more suitable for behavioral experiments, especially for low latency real-time tracking of whiskers. The DVS neuromorphic sensors have been reported to implement real-time low-latency solutions for detecting lines and line segments (Conradt et al., 2009; Grompone von Gioi et al., 2012; Everding and Conradt, 2018), corners (Vasco et al., 2016; Mueggler et al., 2017), and other more complex shapes (Lagorce et al., 2015). Here, we created a virtual spatial area around the mouse’s face and built the “FastEvent” system to track whisker position, and generate feedback based on position of whiskers. This system can be conditioned to track a whisker in real time at low latency. Tracking compares favorably to offline tracking with high-speed cameras. With our system it is possible to generate behaviorally relevant feedback from whisker movement and positioning in 2 ms.

## Materials and Methods

### FastEvent system for tracking whiskers

#### The DVS camera

Here, we use a neuromorphic camera system, the DVS camera (DVS240C; IniVation, RRID:SCR_017283) to track whiskers at a short latency in real time. It consists of a 240 × 180 matrix of sensor units. Each sensor unit contains a set of transistors and a photodiode (Delbruck, 2008; Brandli et al., 2014). Unlike cameras consisting of a CCD or CMOS arrays, each sensor unit uses a differentiator circuit and two comparator units as its output, and each unit generates an ON or OFF event when an increase or decrease in luminance is detected. In contrast to the conventional CMOS/CCD cameras, the DVS camera is frameless; it sequentially collects and transmits information about when and where luminance changes occurred. The biases of each transistor component, i.e., gains and thresholds for luminance changes, in the sensor units can be configured programmatically to adjust the ON/OFF events and ensure that they occur primarily during behaviors of interest. To ensure that event detection occurred rapidly, at short latency, the source-follower bias on the DVS was kept high (Lichtsteiner et al., 2008).

#### Data representation on the DVS camera

**Figure 1.**
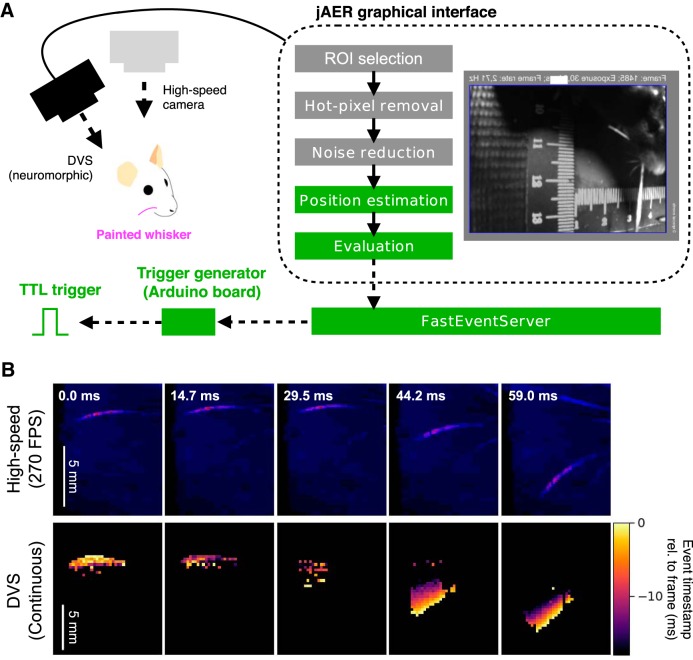
Real-time whisker tracking and triggering with a neuromorphic camera. ***A***, Overview of the FastEvent system. A whisker of an awake head-fixed animal was tracked using a DVS camera. to enhance contrast for *post hoc* and real-time trackings, the tracked whisker was painted with UV paint (magenta) and illuminated with a UV light. The behavior of the animal was recorded with the DVS camera, and a second color camera recording at 200–300 FPS was used as a reference. The data from the neuromorphic camera were sent to the PC in the form of a series of “events” and were then preconditioned. In the preconditioning step, the ROI was clipped, hot-pixels were removed, and noise was reduced with the aid of a graphical user interface, jAER (dotted-line box), that is supplied with the camera. The jAER was customized to estimate object position and generate triggers based on the location of an object (green boxes). Briefly, object positions were estimated by computing weighted averages of the events inside the ROI. The distance between the estimated whisker position and the “target region” (not shown for this figure) was monitored and transmitted to a custom-made independent service program, i.e., the “FastEventServer.” The FastEventServer drove TTL output from a designated board. ***B***, The neuromorphic camera detects motion of the vibrissae. Single representative frames derived from different time points of behavior are shown from a high-speed camera (top panels). For images from a DVS camera (bottom panels), events that occurred within the ∼20-ms period were integrated and displayed. Scale bars = 5 mm.

As a whisker (or any other object) moves in front of the sensor matrix, the field programmable gate array (FPGA) unit on the DVS camera collects the ON/OFF events from the sensor array and converts the events into a format referred to as the address-event representation (AER), a 64-bit binary representation of each event, consisting of the following: (1) a device-internal timestamp; (2) the polarity of the response, i.e., ON or OFF; and (3) *x*- and *y*-coordinates of the source sensor unit, as well as other internal Boolean flags. In short, the read out of the AER provides the location and timing of luminance changes on the matrix of sensors. The series of AER events in the DVS camera were output via a USB 2.0 high-speed connection to the host computer (Lenovo K450e, 3.2 GHz Core i5 4460, with 12 GB DDR3-RAM, 1 TB SATA HDD, and NVIDIA GeForce GT 720, running Windows 8.1 and Java SE 1.8 runtime environment; Fig. 1*A*). The communication between the camera and computer is controlled via a libusb-based driver bundled with the camera. The nominal interval of communication for the host program was configurable and was set to 1 ms, ensuring a low latency.

#### User interface software

The DVS camera is supplied with a Java-based open-source software called jAER (http://jaerproject.org/). Here, we used jAER to modify the transistor biases of the sensor array, to configure the USB communication, and to visualize the spatiotemporal distribution of events coming from the DVS camera. Each iteration of communication with the DVS camera is represented as a packet of events inside the software (Delbruck, 2008). Analytical procedures, being referred to as “filters,” can be used to process each packet and to filter events or generate a new set of events inside the packet. In addition, a set of filters can be chained together to process event packets sequentially one after another.

jAER comes with generic built-in filters, and some were used here: (1) the XYFilter was used to pass events onward if they occur inside a user-specified region of interest (ROI; Fig. 1*A*, ROI selection); (2) the HotPixelFilter was used to remove the events generated on sensor units that produce a large number of dark events (Fig. 1*A*, hot-pixel removal); and (3) the BackgroundActivityFilter was used to remove solitary events, i.e., when a single sensor unit generates an event and no adjacent units emit an event at the same time (Fig. 1*A*, noise reduction).

In addition to using the generic built-in filters, we designed a custom-made MeanTracker filter that tracked whisker position. This filter was used to estimate positions of a labeled object (Fig. 1*A*, position estimation). The MeanTracker assumed that there was only a single luminescent object in the field of view, and computed this object's position by calculating the center of mass of the detected object. Using a weighted-average scheme, the MeanTracker updated the estimated position of the object each time an event packet was received.

More specifically, the MeanTracker filter computed the weighted average of the i-th event packet, x_i_, based on the recorded positions of individual events in the the i-th packet, {x_ij_} (j = 1, …, N), as:

$$x_{i}=\frac{1}{N}\underset{j}{\sum }x_{ij}.$$

For each time point t, the weighted contribution of the i-th packet w_i_, whose reception time was t_i_, was computed as:

$$w_{i}=e^{\frac{t_{i}-t}{\tau }},$$

where τ is the integration time constant. By using pairs of the weight w_i_ and the position x_i_, the position of the object, x_e_(t), at each time point t, was estimated as a weighted average of the contributions of all the packets up to the time point:

$$x_{e}(t)=\frac{\sum_{i}w_{i}x_{i}}{\sum_{i}w_{i}}.$$

We introduced this weighted-averaging approach because the number of events in each packet varied considerably. When the number of packets is too small, noise events coming from unlabeled objects contribute inordinately in the event packet, and can lead to inaccurate position estimates. We therefore used a short 200- to 300-μs integration time constant τ to ensure that at least tens of events contribute to position estimation even when not much movement was observed. Real-time estimation of labeled–object positions was output to a comma separated variable (CSV) file, referred to as the MeanTracker log which was saved for *post hoc* analysis.

#### Virtual target-based position evaluation

The position of the tracked object computed by MeanTracker was evaluated (Fig. 1*A*) by specifying a rectangular “virtual target” region on the jAER graphical interface, and by assessing whether the tracked object position was inside or outside of the target region. The virtual target region was interactively specified throughout a single recording session. The status of the evaluation was also logged to the MeanTracker log file. The coordinates of the virtual target region were logged to another CSV file and were referred to during analysis.

#### Generating feedback

The output from the MeanTracker was a TTL-high or TTL-low level, reflecting the position of the tracked object, i.e., whether it was inside or outside the virtual target region. Through a UDP communication, the resulting evaluation was output to a custom C++-based program called the FastEventServer. This server, which ran on the same PC that ran the jAER, commanded the output to a driver program (Fig. 1*A*).

The output driver for FastEventServer was based on the Arduino UNO single-board microcontroller (https://www.arduino.cc/, RRID:SCR_017284). It used an ordinary USB-serial communication that is built into the Arduino UNO (i.e., a serial communication over a USB 1.1 full-speed connection). Because the original UNO kernel (also known as bootloader) cannot work at rates that are >1 kHz, we implemented a custom kernel, which we call the arduino-FastEventTrigger kernel, using LUFA (http://www.fourwalledcubicle.com/LUFA.php; release 100807), based on the Arduino-usbserial project (https://github.com/arduino/ArduinoCore-avr/tree/master/firmwares/atmegaxxu2/arduino-usbserial). The Arduino-FastEventTrigger kernel has the Atmega16u2 USB-to-serial chip (instead of the main ATmega328P chip on the UNO) generating the output in response to the USB–serial commands.

#### Hardware for detecting the timing of the triggered output

To determine the response latency of the FastEvent system, we used the auxiliary TTL-level digital input port equipped on the DVS camera. The FPGA unit on the camera treats the edges of TTL signals as “special events” and gives them timestamps, as it does for the sensor ON and OFF events. By plugging the output of the FastEvent sytem to this TTL input port, it was possible to estimate the total response latency by examining the sensor event timestamps and comparing them to the special event timestamps. The difference in the timing of these events corresponded to the delay between the onset of motion and the output of the FastEvent system.

#### Set-up specifications

The DVS camera was used with a Kowa LM5NCR objective (f = 4.5 mm, F1.4 with manual iris; the iris was fully or almost fully opened during the experiments). The resulting field of view for the DVS camera was ∼40–50 mm. The ROI of tracking was selected so that it covered the full-sweep of the UV-painted part of the whisker, and was typically 20–40 pixels (3–5 mm) along the mediolateral axis and around 100 pixels (∼15 mm) along the anteroposterior direction. The Arduino-based output board was connected to the computer with a USB 1.1 cable. A BNC connector was attached to the output of the Arduino-based board and this output was connected directly to the DVS camera and other data acquisition devices.

### Experiments using a plucked whisker

#### Preparation

A B1 whisker was plucked from an anesthetized mouse. The whisker was glued to the arm of a servo motor (Futaba S3114) and painted using the UV dye (see the section below). The servo motor was driven by an Arduino UNO board, by means of the “Servo” library (https://www.arduino.cc/en/Reference/Servo). The motor swept the whisker 12° back and forth in 1–6° step. The interval between steps was 20–300 ms. The whisker was driven at different frequencies, in a particular sequence from a low frequency to a high one: 0.139 Hz (1°/step, 300-ms clock cycle, five sweeps), 0.417 Hz (1°/step, 100-ms clock cycle, five sweeps), 1.04 Hz (1°/step, 40-ms clock cycle, five sweeps), 2.08 Hz (1°/step, 20-ms clock cycle, 10 sweeps), 4.17 Hz (2°/step, 20-ms clock cycle, 20 sweeps), 6.25 Hz (3°/step, 20-ms clock cycle, 20 sweeps), 8.33 Hz (4°/step, 20-ms clock cycle, 40 sweeps), and 12.5 Hz (6°/step, 20-ms clock cycle, 40 sweeps).

#### Data acquisition

The sequence of motion was repeated several times using different integration time constants for the MeanTracker on the FastEvent system. The DVS camera was positioned for a top view, capturing the back and forth rotation of the whisker. As a check of the actual motion of the motor and whisker, a Basler acA800-510uc high-speed camera was deployed at 200 FPS. In addition to the computer running the FastEvent system, another Windows 10 computer was used to run a custom-made acquisition software (ZR view, Robert Zollner, Eichenau) to acquire high-speed videos.

#### Analysis

We estimated the angle of whisker rotation, individually from the high-speed videos and from the MeanTracker log files generated from the FastEvent system, before comparing the two data types.

To track whisker motion on the high-speed videos, the “videobatch” python library was used. In this analysis we used the data acquired at a lower frequency (0.139 Hz) and fit the commanded movement of the whisker to the actual motion recorded with the high-speed camera. The result of this fitting process was used to estimate the angle of rotation.

For the FastEvent tracking data, the center of rotation in the field of view during each trial was first estimated by visual examination of the two-dimensional movement histograms derived from the raw AER data. The radius and the angle of rotation at each time point were then computed based on the *x*- and *y*-coordinates recorded on the MeanTracker log file.

To correct for the different time bases for high-speed camera tracking and the FastEvent tracking, the two data streams were temporally scaled. Briefly, we first split the data into periods of individual frequency settings. Then we manually marked the first and the last turns in each frequency setting for both high-speed and FastEvent tracking streams. Finally, we fit the periods between the two time points with a least-squares approach. The RMS error was defined as the moment-to-moment absolute difference between the fitted horizontal motion based on the high-speed video and the horizontal motion from the MeanTracker log file (i.e., FastEvent tracking). The gain of motion was defined as the ratio of the SD for the motion on FastEvent tracking and SD of the motion tracked in the high-speed video.

### LED-based experiments for estimating the trigger latency

Two LEDs (588 nm, yellow, 5 mm in diameter) were positioned side by side, ∼15 mm apart, and their flickering was captured in a top view by the DVS camera. We configured the view so that only one LED was located inside the target region. The on/off sequences of the LEDs were controlled by an Arduino UNO so that their flashing alternated at 4 Hz (250 ms/cycle). To minimize the effect of the integration time constant on the FastEvent tracking, each LED was flashed only for 62 ms (i.e., 1/4 cycle) of every cycle. Because the event rate can affect the processing latency, the brightness of the LEDs was tuned so that the average DVS event rate matched the event rate during the animal experiments (i.e., 10–50 events/ms). The on/off command signals for the LEDs, and the output triggers from the FastEvent system, were recorded at ∼16.6 kHz using the Power1401 interface (CED, RRID:SCR_017282) and the Spike2 software (CED, RRID:SCR_000903).

### Animal preparation

All animal procedures were performed in accordance with protocols approved by Charité–Universitätsmedizin Berlin and Berlin Landesamt für Gesundheit und Soziales (LaGeSo) for the care and use of laboratory animals.

#### Animals

C57 black six mice (*n* = 5; RRID:IMSR_JAX:000664) were used in this study. Mice were used for validation during system and behavioral task development (i.e., profiling the tracking and triggering) and were trained in a behavioral task. Animals were housed under the 12/12 h reverse light/dark cycle.

#### Surgery

A headpost was surgically attached on the skull. Animals were anesthetized with ketamine/xylazine (90/10 mg/kg body weight) and placed on a feedback regulated heating pad. After subcutaneous lidocaine injection, skin and fascia were removed. A lightweight aluminum headpost was attached using a Rely-X (3M) cement, followed by Jet acrylic black cement. Animals were monitored during recovery and were given antibiotics enroflaxicin and analgesics (buprenorphine/carprofen).

#### Whisker painting

UV painting of a whisker increased its reflectance and helped tracking, in both the *post hoc* analyses of high-speed videos (Nashaat et al., 2017; Dominiak et al., 2019) and in the real-time DVS tracking by amplifying the signal-to-noise ratio to the other non-painted objects. At the beginning of each recording session, a small amount of UV paint (UV glow) was applied on the C1 whisker. During data acquisition, a custom powered, inhouse UV-LED torch, was used to deliver UV-A (370–400 nm) illumination that made the painted whisker glow. The UV torch generated a beam with 3- to 3.5-cm diameter, with ∼300 mW of power, and a radiance of ∼10°. The torch was positioned 20–25 cm above the animal, with the beam directed from behind the animal, toward the painted whisker (for more details, see Nashaat et al., 2017).

### System profiling experiments

For profiling tracking and triggering using the FastEvent system, we tracked a labeled whisker in naïve mice, i.e., those that had not been trained in any specific behavioral task with their whiskers. After surgery, mice were habituated to the experimenter, to the setup, to head-fixation, and to whisker painting, before being used in the experiments. Three to 4 d of handling, head fixing, and painting were sufficient to acclimate them. During acquisition, the animal was head-fixed, and its whiskers were tracked under UV illumination.

The DVS camera and a Basler acA800-510uc high-speed color camera were positioned above the animal. High-speed videos (250–270 FPS) and FastEvent system data were acquired simultaneously. Two data acquisition computers were used; one was used to run the FastEvent system, the other Windows 10 computer was used to acquire the high-speed video with the ZR view inhouse acquisition software. The output of the FastEvent system, i.e., the output of the Arduino-based board, was monitored as an input in the auxiliary input port of the DVS camera (see the descriptions above for details).

### Sensory feedback experiments

We performed a visual association experiment, where mice were trained to lick after a flash of an LED during the auditory Go-cue. During training and during the experiment, mice were water deprived up to 85% of their initial body weight. Mice were first trained to perform a visual detection task in which a visual cue (800-ms duration, flashing at 2–5 Hz) was presented in their field of view, at random intervals. Visual stimuli were generated using a green (568 nm) LED and were delivered through a fiber optic cable.

The intervals between stimuli were exponentially distributed with a mean value of 8–10 s. Mice obtained a reward if they responded to the cue by licking during the response window which began 3 s after the onset of LED flashes. To suppress spontaneous licking behavior, a time out punishment was imposed; if a mouse licked in the second before the visual stimuli began, the onset of the next visual cue was delayed, i.e., there was increase in the waiting period. The hit rate and the rate of spontaneous licking were used to assess the animal’s performance. For monitoring the licking behavior, a piezo element was attached to the lick port, and its signal was amplified using an inhouse custom made amplifier. A drop of water (2–5 μl) was used as the reward, and its delivery was controlled using a solenoid valve (Takasago PS-1615NC).

Once the animals had learned the association between the visual cue and the reward, the Passive behavioral paradigm began. In this paradigm, mice were trained to lick in response to the visual cue (800-ms duration, flashing at the 2- to 5-Hz frequency) when an auditory cue, i.e., a cue that turned on before the visual cue, was also audible. The auditory Go-cue was generated using a piezo buzzer, and was delivered intermittently at 3–5 Hz for up to 5 s. The latency to the visual cue after the auditory cue turned on was variable but it was at least 250 ms and up to 2.5 s. The auditory cues were terminated right after the animal licked irrespective of whether the visual cue had already been presented or not. Mice were rewarded only when they licked after the visual stimulus was flashed on. Intervals between individual auditory Go-cues were set randomly (exponentially distributed with 8–10 s mean), but as described above, they were reset if the animal licked <1 s before the beginning of the auditory cue.

After several sessions in which mice performed the Passive behavioral paradigm, we trained mice in an Active paradigm. Mice had to perform almost the same task in both the Passive and Active paradigms, except that in the active task, the visual stimulus was generated as a sensory feedback. Based on the output from the FastEvent system, the LED turned on when the painted whisker was inside the virtual target region. Mice were rewarded if they licked after the LED turned on, i.e., the painted whisker was moving into the virtual target region, and the auditory Go-cue (delivered intermittently at 3–5 Hz for up to 5 s) was also active. Note that once the cross-threshold event was triggered during the cued period, the animal could move its whiskers out of the target region. During licking, there was no requirement for the whiskers to be at any particular target region.

#### Hardware

Licking, whisk events, as well as multiple other tasks were monitored, controlled and stored using a single-board computer (STM32 NUCLEO-F411RE, ST Microelectronics) which in turn was controlled and monitored by a PC with a Python program based on the “ublock” custom library.

A Basler acA800-510uc high-speed camera was used to acquire videos at 200 FPS, by means of the ZR View acquisition software running on a Windows 10 computer. Data acquisition was configured such that a single trial of video frames centered around reward delivery, i.e., 2.5 s before and 2.5 s after the reward, were saved as individual movies. The timing of the sensory cues, reward, the TTL output from the FastEvent system, and the beginning and end of each trial were also captured with Spike2 software, via a 1401 data acquisition interface.

### Analytical procedures

#### Experimental design and statistical analysis

For each animal experiment, we used two adult (at least 60 d old) male mice with the C57B6 background. We ran four behavioral sessions for the system profiling (i.e., no-task) experiments, and 13 behavioral sessions (two Passive, and 11 Active) for the experiments with behavioral tasks. For the Active paradigm we acquired and analyzed 1479 trials, and for the Passive paradigms, we used 337 trials. We used Kolmogorov–Smirnov (KS) test to examine differences.

During examination of whisking strategy under the Active paradigm, each of the 11 behavioral sessions (five to six sessions each from the two animals) consisted of at least 10 trials out of at least five target locations. To determine whether the univariate linear regression model had a non-zero slope, we used Mann–Whitney *U* test. To determine whether the multivariate linear regression model had a significant explained variance, we compared the results of the raw dataset with the outcomes from the corresponding shuffled dataset, using Wilcoxon signed-rank test. To compare the amount of variance explained by the set point and by the amplitude during each session we used the Wilcoxon signed-rank test. To compare the variance explained in the different trial phases (i.e., Wait, Hit, Lick; see below for definitions) in each session we used a Wilcoxon signed-rank test with Holm–Bonferroni correction.

#### Software

Positions of the labeled whisker in high-speed videos were tracked using a custom-made ImageJ (RRID:SCR_003070) plugin (“Pixylator”) or the equivalent “videobatch” custom Python library. These libraries work in a similar fashion as the real-time tracking on jAER does, i.e., they compute the weighted average of luminance intensities of the region with similar hue values (Dominiak et al., 2019). To generate event data at arbitrary time points the AER-format log files generated by jAER were read and analyzed in Python, by using the “aerpy” custom library.

Python (https://www.python.org/, version 3.7.2, RRID:SCR_008394; van Rossum, 1995), Scipy (http://www.scipy.org/, version 1.2.0, RRID:SCR_008058; Jones et al., 2001), NumPy (http://www.numpy.org/, version 1.15.4, RRID:SCR_008633; van der Walt et al., 2011), Bottleneck (https://github.com/kwgoodman/bottleneck, version 1.2.1), Matplotlib (https://matplotlib.org/, version 3.0.2, RRID:SCR_008624; Hunter, 2007), Pandas (https://pandas.pydata.org/, version 0.23.4; McKinney, 2010), and Jupyter (https://jupyter.org/, version 1.0.0; Perez and Granger, 2007) were used for general analytical procedures.

#### Alignment of DVS and high-speed camera data

The spatial and temporal scaling of the DVS camera system varied from experiment to experiment. When the system was being tested and profiled, the time course of whisker positions in each MeanTracker log file was first aligned to the corresponding high-speed video data by matching the manually-selected representative whisker positions that were evident as peaks in a trace of whisker movement. A linear regression between the two sets of temporally-aligned whisker positions (in the high-speed video and in DVS) was then performed to determine the spatial scale of the DVS compared to that of the high-speed video. To determine the scale of the DVS during training sessions, histograms of the whisker position acquired with the high-speed videos and in the DVS MeanTracker logs, were compared. In particular, the curve of each MeanTracker histogram was fit to its high-speed video counterpart by linear transformations of the position and the log-fraction, by minimizing the squared-error in the direction of the log-fraction. The resulting coefficient for transformation was used to estimate the target positions for the whisker.

#### Behavioral events

Lick and threshold crossing (whisk) events were extracted from the session log file generated by the ublock Python library during the behavioral task. To calculate success rates, trials in which mice were rewarded were considered a “success.” Trials were discarded, unless they included one whisk event during the auditory cue period, or unless the mouse responded to the auditory cue with a lick. In the calculation of whisk event frequency, we performed a *post hoc* debouncing procedure, so that neighboring whisk events had a minimum interevent interval of 20 ms.

#### Timestamp-based estimation of trigger latency

We examined the difference in device-internal timestamps between the sensor events (i.e., when the motion occurred) and its special events counterpart (i.e., when the TTL output was delivered). To do so, we picked up the first event packet that contributed in changing the trigger status, i.e., the first event packet after the estimated whisker position crossed the border of the target region. The earliest event timestamp in the event packet was considered to be the timestamp for the event packet, and thus were the time point of motion event generation (i.e., trigger evaluation). We then identified the corresponding special event in the series of events in the AER log file: because special events occur when the DVS camera detects edge signals in its auxiliary input, this event should correspond to the physical occurrence of the trigger. For each border-crossing events, we computed the difference between the timestamp of the motion-derived event packet and the timestamp of the corresponding special event.

#### Analysis of whisker dynamics

Upper and lower bounds including the entire envelope of whisker position were calculated based on the minimal and maximal values within a 100-ms radius sliding window. To minimize the effect of any abrupt changes, the results of the sliding-window analysis were further smoothed using the 100 ms-radius sliding-mean filter. Lower bound of the envelope was then defined as the (instantaneous) set point. The difference between the upper and the lower bounds of the envelope was defined as the (instantaneous) amplitude.

For the analysis of trial-aligned whisker motion, we split each trial into three periods: a Wait (–1.5 to –0.5 s relative to the reward trigger), Hit (–0.5 to 0 s), and Lick (0 to +1 s) periods. The lower and upper bounds of whisker movement, as well as the amplitude, were averaged for each period, and each trial. To estimate the slope of each whisking parameter, with respect to the target location, we performed a linear regression.

To estimate the contribution of set points and amplitudes to the variability of target positions, a multiple linear regression model was built. For each trial-based period *p* for all trials from each behavioral session, we solved a regression problem to estimate contribution for the variability of set points, *X*_setpoint_, and amplitudes, *X*_amplitude_, to the variability of target positions, *X*_target_, for individual trials:

$$X_{target}∼A_{p}X_{setpoint}+B_{p}X_{amplitude}+C_{p},$$

where the subscript *p* stands for one of the Wait, Hit, and Lick trial-based periods. For all the positional values, the averages during corresponding trial-based periods were used. After estimating the optimal parameter set {*A*_p_, *B*_p_, *C*_p_}, the residual variability was computed by subtraction of the estimated target position (i.e., from the set point and the amplitude of the trial), ${\hat{X}}_{target}$, from the actual target position $X_{target}$ to be summed up as:

$$SS_{data}=\underset{trial}{\sum }{\left(X_{target}-{\hat{X}}_{target}\right)}^{2}.$$

The *R* ^2^ values, *R* ^2^_data_, were calculated from *SS*_data_ and the total variance of the target, $SS_{total}=\sum^{\;}{\left(X_{target}–E(X_{target})\right)}^{2}$, as *R* ^2^_data_ = 1 – *SS*_data_/*SS*_total_. For the shuffled dataset, regression and the rest of calculation were performed using the shuffled target positions to derive *R* ^2^_shuffled_. Wilcoxon signed-rank test was used to compare the statistics between *R* ^2^_data_ and *R* ^2^_shuffled_ for each trial-based period. For multiple pairwise tests between different trial-based periods, Wilcoxon signed-rank test was performed with Holm–Bonferroni correction.

To estimate the contribution of set points and amplitudes, the data sets were partially shuffled. In the case of set points, regression analysis was performed on the set point values after they had been shuffled. The variability with shuffled set points, *R* ^2^_*setpoint_, was first computed. The contribution of the set point was then estimated as *R* ^2^_data_ – *R* ^2^_*setpoint_. The same procedures were followed in the case of amplitude. Wilcoxon signed-rank test was used to compare contributions of set points and amplitudes for variability of targets. For multiple pairwise tests between different trial-based periods, Wilcoxon signed-rank test was performed with Holm–Bonferroni correction.

#### Code availability

The software described in the paper is freely available online at https://github.com/viktorbahr/jaer (FastEvent modified jAER, version 0.3.1), https://github.com/gwappa/arduino-fasteventtrigger (Arduino board-based trigger output generator, commit 4b0790f), https://os.mbed.com/users/gwappa/code/STM32_Whisking/ (STM32 nucleo-based task controller, revision 32:1416e015016c), https://github.com/gwappa/python-ublock/ (the ublock task-control/monitor program, version 0.1.1), https://github.com/gwappa/Pixylator (Pixylator color-tracking plugin for ImageJ, version 0.5), https://github.com/gwappa/python-videobatch (videobatch batch-tracking program, version 1.0), https://github.com/gwappa/python-aerpy (aerpy, commit 694d5ca).

## Results

### Implementation of neuromorphic camera-based real-time tracking

Proof of principle experiments in five mice were performed with the customized neuromorphic camera system. To determine whether single whiskers could be tracked using the DVS camera, the whisker was painted with UV paint, backlit and tracked with a conventional camera-based tracking approach (Dominiak et al., 2019), and with a neuromorphic camera (Fig. 1*A*). Once the sensitivity of pixels had been tuned, the labeled whisker could be clearly detected by the DVS camera and simultaneously by the high-speed color camera (Fig. 1*B*).

Next by collecting packets of motion events, i.e., luminance changes above a certain threshold in individual pixels, an object-tracking algorithm was implemented by integrating events over time. To reduce the effect of “noise,” i.e., to remove events that did not derive from the labeled whisker, and to account for variability in the number of events in individual packets, each packet was weighted with an inverse exponential of the time elapsed (for details, see Materials and Methods).

**Figure 2.**
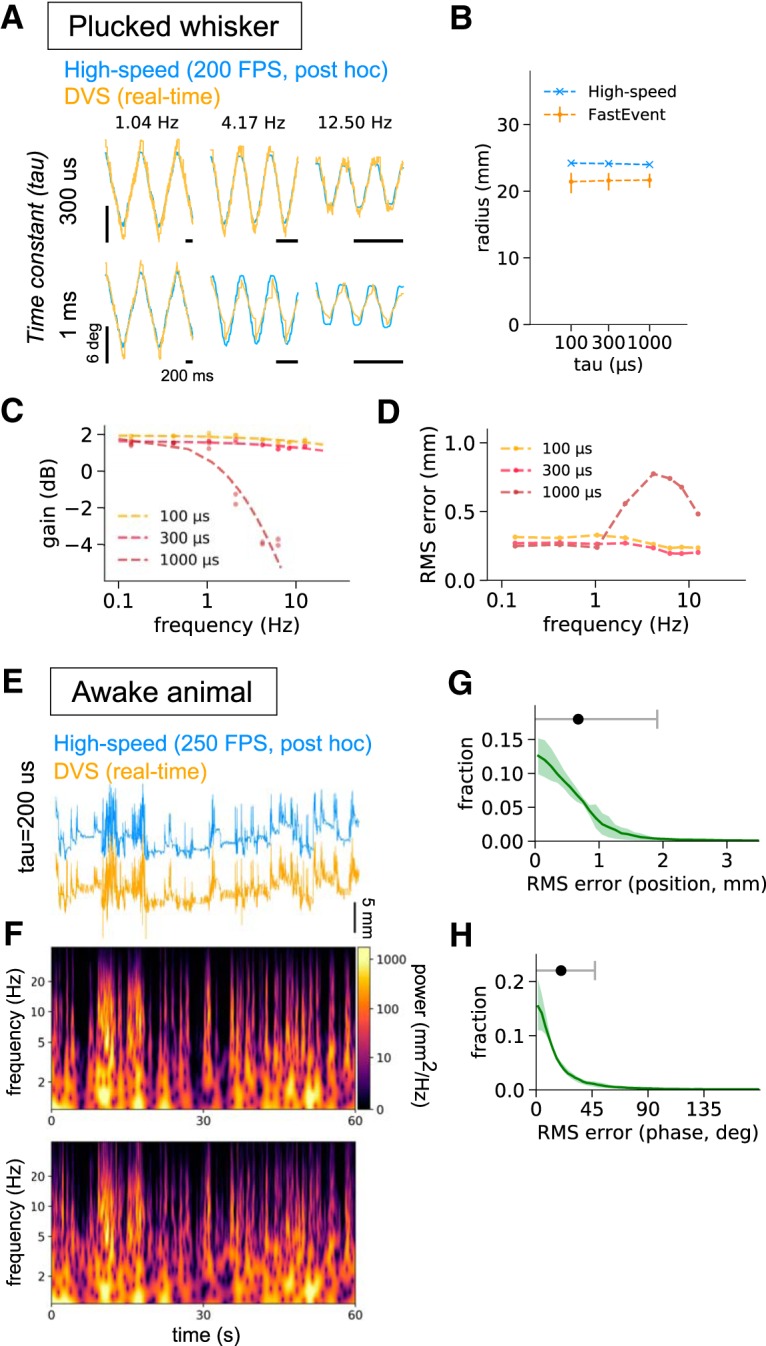
Real-time whisker tracking in artificial settings and in awake mice. The motion of a plucked whisker attached to the arm of a servo motor sweeping at ∼0.1 to ∼12 Hz, was tracked using a high-speed camera *post hoc* tracking (blue) and was simultaneously tracked with the FastEvent real-time tracking system (orange) using different integration time constants. ***A***, Representative angular positions from different sweep frequencies. Data are shown for 300-μs and 1-ms integration time constants. ***B***, The radii of motion detected by both tracking methods. Error bars for the FastEvent system corresponding to the 2.5th and the 97.5th percentiles of the data. ***C***, Effects of changing the integration time constants on the gain of the angular motion detected with the real-time system. The regression curves for 100-, 300-, and 1000-μs integration time constants are generated by fitting Butterworth filter characteristics to the observed values. ***D***, Effects of integration time on RMS error. The moment-to-moment positional RMS error was averaged and plotted for individual sweep frequencies, and for each integration time constant. Once the real-time FastEvent system settles on a correct frequency of motion, it can track whisker position at ∼0.3-mm accuracy. ***E–H***, Tracking of whisker motion from awake animals. ***E***, Whisker-position traces were derived from a high-speed camera (blue) and the DVS neuromorphic camera (orange) in awake head-fixed mice. Whisker positions were recorded simultaneously with both cameras for 1 min and aligned *post hoc*. ***F***, Wavelet (i.e., time-varying) power spectra. The offline high-speed camera and the neuromorphic camera tracked the whisker similarly, suggesting that the DVS camera tracked whisker dynamics precisely in real time. The power spectra were generated from the traces shown in ***E***. ***G***, Plot of the RMS error for the offline and real-time data. The medians and 2.5th and 97.5th percentiles across four sessions are shown. The black point above the plot represents the grand average (mean), and the error bars are the SDs, 0.674 ± 1.235 mm. ***H***, Plot of the difference in phase for data analyzed offline and acquired in real time. The plot represents the mean ± SD in phase differences across the 1- to 30-Hz frequency range. Histograms from four sessions (two sessions each for two animals) were averaged. The black point and the gray error bars indicate the grand average and SDs, 20.0 ± 27.3°.

To profile the real-time tracking efficiency of this system, we performed experiments on a plucked B1 whisker driven by a servo motor. To examine the effect of integration time constants on the real-time tracking, we used three different values for the time constant: 100, 300, and 1000 μs (Fig. 2*A–D*). An integration time constant of 300 μs was almost as effective as the high-speed camera-based *post hoc* tracking (Fig. 2, top panels). Nevertheless, the real-time-tracked trace appeared noisier, and overshot the *post hoc* tracking especially at times when the whisker changed directions. These differences in the tracking results seem to reflect the differences in the tracking strategies. The FastEvent system is based on tracking of the leading edge of motion, whereas the high-speed camera-based tracking is based on the center of mass of the object.

Increasing the integration time constant to 1 ms decreased the apparent noise in the traces (Fig. 2*A*, bottom traces), possibly because of the increased number of events per estimation. The observation that increasing the integration time constant reduced the variability in the observed radius of motion during tracking (Fig. 2*B*) and led to smaller tracking errors at low frequencies (Fig. 2*D*) support this possibility. But there were negative consequences of increasing the time constant to 1 ms: it created a low pass-filtering effect on the estimate of position (Fig. 2*A*,*C*), which led to larger errors in the frequency domain corresponding to whisking (>5 Hz; Fig. 2*D*). Taken together, these observations suggested that a 200- to 300-μs integration time constant for the FastEvent system was the most stable for tracking frequencies up to 30 Hz. The angular errors were almost stable at ∼1°, resulting in ∼0.3-mm positional error for the three integration time constants here (Fig. 2*D*).

When we used awake, head-fixed mice that had not been trained in a task, and tracked their whiskers, the data from the real-time tracking of whisker position and *post hoc* tracking of the same trials and sessions generated comparable data (Fig. 2*E*). The moment-to-moment difference in position estimated by the two methods was ∼1 mm (Fig. 2*G*).

Analysis of the time varying power spectra of whisking showed that both the offline high speed (Fig. 2*F*, top) and the DVS camera (Fig. 2*F*, bottom) captured the same frequency components. The statistics of the differences in the data acquired by the DVS and offline high-speed camera show that the majority of the data acquired in the two data streams fell between ±15° of each other (Fig. 2*H*). Together, these results demonstrate that the DVS neuromorphic camera can be used to track single mouse whiskers in real time.

### Generating feedback in real time

**Figure 3.**
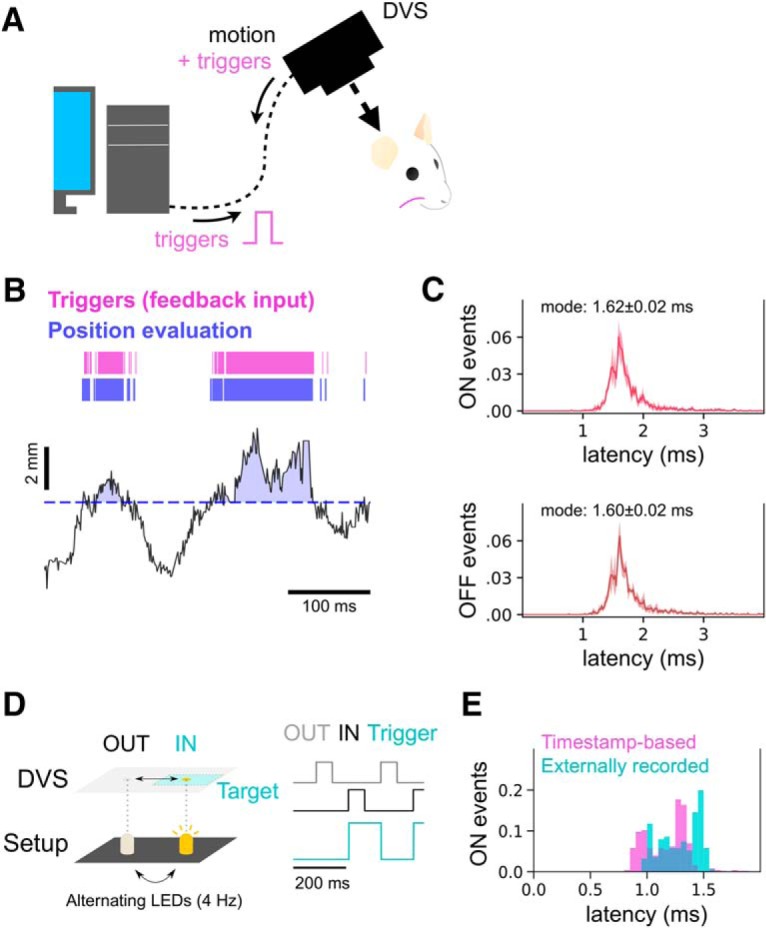
Real-time, low-latency triggers generated by whisker position. ***A***, Schematic of the behavioral experiment. Whisking behavior of awake, freely-whisking head-fixed animals was recorded using the DVS camera. After processing on the host computer, TTL pulses were generated based on whisker position. These pulses were injected back into the auxiliary TTL port of the DVS camera (magenta), which in turn reported pulses on the same time base as the motion-related events. ***B***, Comparison of internal trigger (blue) and actual trigger generation (magenta). Whisker positions were estimated in real time and are shown at the bottom (black, solid line). An arbitrary position threshold (blue, dotted line) was set for the session, and at any location above this position a TTL-high level was generated (region shaded blue). To estimate the round-trip latency, the timing of the output detected by the program (position evaluation, blue rectangles) and the timing of the TTL pulses detected at the DVS camera (triggers, orange rectangles) were compared *post hoc*. ***C***, Timestamp-based estimation of round-trip latency, plotted as histograms. Latency distributions for ON events (the whisker going into the target, TTL low to high; top panel) and OFF events (going out of the target, TTL high to low; bottom panel) are examined separately. Most of the triggers were generated within 2.5 ms (mode of histograms, ∼1.6 ms). ***D***, External recording-based estimation of trigger latency. To generate exactly timed motion, two LEDs flashed in alternations at 4 Hz (left, bottom). This flickering was captured by the DVS (left, top). Command signals to the LEDs (right, gray and black traces) and generated triggers from the DVS (right, blue traces) were recorded. ***E***, Comparison of timestamp- and external recording-based estimation of trigger latency. Because of the latency to luminance event generation, timestamp-based estimation resulted in slightly shorter latencies (*N* = 502 ON events; timestamp-based, 1.19 ± 0.23 ms, external recording-based, 1.29 ± 0.17 ms).

The goal of real-time tracking of behavior is to provide feedback to the animal, i.e., to manipulate the environment around the animal or to optogenetically manipulate the brain. To achieve this goal, we implemented an output from the DVS camera system that tracked whisker motion to a virtual target in real time (Fig. 3*A*). Whenever the whisker was within the virtual target region (Fig. 3*B*, blue), an output event was triggered (Fig. 3*B*, pink). The latency to trigger an output was measured by taking the output (a TTL pulse) when the whisker moved to a target position and feeding it back directly into the DVS camera (Fig. 3*A*,*B*; Movie 1). We found that the TTL signals fed back to the DVS camera closely tracked the estimated whisker position, and they reflected whether the whisker was in the virtual target region (Fig. 3*C*). Feedback to the DVS camera when the whisker entered or exited the target region occurred at around ∼1.6 ms (Fig. 3*C*; Movie 1).

Movie 1. Real-time tracking of whisker positions of a freely whisking mouse. The setup is as described in Figure 2. During the ∼3-min session, the animal was head-fixed and allowed to whisk freely under a high-speed (270 FPS) and a neuromorphic (DVS) cameras. The crosses indicate the estimate of whisker position, the dotted line indicates the virtual target position. Changes in colors of signs from white to magenta shows the TTL signal generated from the FastEvent system, as it was fed back to the DVS camera (i.e., the timing of real-time feedback). Information from the DVS camera was aligned and then down sampled to annotate the high-speed video. A 5-s period was used to generate the video. 10.1523/ENEURO.0147-19.2019.video.1

Although our method, based on internal timestamps, is easy to use and provides an estimate of the trigger latency, it does not provide an estimate of the latency from the time point when an object moved. Because it was difficult to know the exact timing of whisker movement and the subsequent output/generation of a trigger, we used another electronic approach. We measured the motion created by flashes of a pair of LEDs turning on and off. Motion was captured by the DVS camera, and the timing of trigger generation measured against the flash of light from the LED (Fig. 3*D*). When we use this method to estimate the latency, a miniscule ∼0.1 ms, over and above the latency estimated based on the internal time stamps method, was added to the value of the latency estimate (Fig. 3*E*; *N* = 502 ON events; timestamp based, 1.19 ± 0.23 ms, external recording based, 1.29 ± 0.17 ms). The additional 0.1 ms probably arises from the processes inside the DVS camera that generate and timestamp an event. This latency of event generation was consistent with the earlier reports on the DVS system (Lichtsteiner et al., 2008; Brandli et al., 2014). Taken together, our system can track motion and generate feedback signals at low latency below 2 ms.

### Mice can associate the real–time feedback with whisker positions

**Figure 4.**
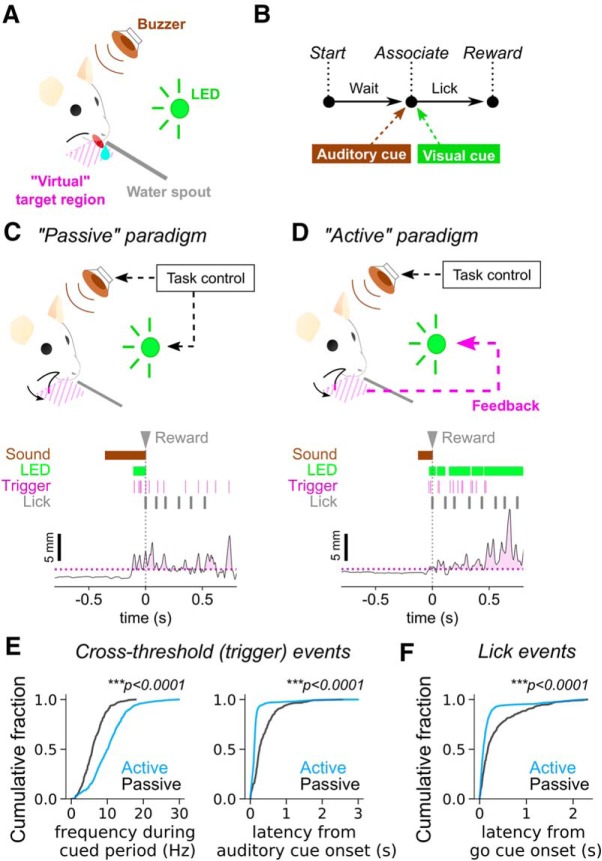
Mice associate sensory feedback with whisking. ***A***, Schematic of the setup. The mouse was head-fixed with one of its whiskers being dabbed with UV paint. A buzzer (brown) was placed around the setup, and a 568-nm LED (green) was set in the animal’s visual field. The water spout (gray) was positioned to deliver water reward and to monitor the animal’s lick behavior. A virtual target region (magenta) was placed around the sweeping region of the painted whisker. The position of the virtual target was adjusted during individual sessions using the FastEvent system. ***B***, Behavioral task. The task is a multisensory association task, where the animal waits until it receives both auditory (brown) and visual (green) cues at the same time. The animal reports its sensation by a lick on the spout, which triggers reward delivery (2- to 5-μl water). ***C***, Passive paradigm. In the passive paradigm, both auditory and visual cues were controlled by the task controller. In this paradigm, whisk events (trigger, magenta), generated when whisking behavior reached the target position, were monitored and recorded, but were not reported back to the animal. Sensory cues, trigger events and licks are shown in the raster display above the trace of whisker position. ***D***, Active paradigm. In the active paradigm, the visual cue is generated by feedback of whisk events (trigger) when the painted whisker goes into the virtual target region. The auditory cue is controlled by the task controller. Whisker positions in ***C***, ***D*** across sessions and trials were aligned by their base whisker position (the median position of the whisker during the initial 30 s of each session without engagement to any behavioral tasks). ***E***, Profiles of threshold crossing (whisk) events based on whisker-position. Events that triggered an output event during successful trials are selected and trial-wise distributions of frequency of whisk events during the auditory-cued period (left) and the latency to the initial whisk events from the onset of the auditory cue (right) were plotted as cumulative histograms. For calculation of frequency, we first performed *post hoc* debouncing procedures so that whisk events had an interval of at least 20 ms. Only trials with more than two whisk events were used for this analysis. The average interevent intervals were inverted to compute the event frequency. Compared to those in the passive paradigm (black), trials in the active paradigm (blue) were found to have significantly more frequent threshold crossing events (*p* = 1.32 × 10^−26^, KS test; *N* = 236 passive vs 646 active trials from six to seven sessions each for two animals), with a lower latency to the auditory cue onset (*p* = 2.81 × 10^−74^, KS test; *N* = 337 passive vs 1479 active trials from six to seven sessions each for two animals). ***F***, Latency of lick response from the onset of the visual cue during the auditory-cued period of successful trials. Cumulative histograms were generated for the active (blue) and the passive (black) paradigms. The latency was significantly smaller for trials in the active paradigm than those in the passive paradigm (*p* = 8.84 × 10^−23^, KS test; *N* = 337 passive vs 1479 active trials from six to seven sessions each for two animals).

Can mice be trained to move a particular whisker to a target area, and can they make use of real-time feedback from the whisker position? To examine whether feedback could be used by mice, we trained two mice to whisk toward a virtual target (Fig. 4*A*,*B*). An auditory cue initiated the trial and it stayed on for 5 s, or until mice licked. Correct positioning of whiskers in the rectangular target region activated the visual stimulus, i.e., a fiber optic connected to an LED placed in front of the mouse (for detailed descriptions, see Materials and Methods).

To examine the effect of training to move to a virtual target, we compared the whisker positions in the passive task (Fig. 4*C*), to whisker position in an active task (Fig. 4*D*). In the passive paradigm, whisker position was not associated with a target, but the LED indicated that mice could lick. Compared to the Passive task (Fig. 4*E*, black), during the Active task (Fig. 4*E*, blue) mice positioned their whiskers to generate whisk events, i.e., threshold-crossing events, significantly more often during the cued period (*p* = 1.32 × 10^−26^, KS test; *N* = 236 passive vs 646 active trials; Fig. 4*E*, left). In addition, the latency from auditory cue onset to a whisk event (Fig. 4*E*, right), and the latency from the visual cue onset to a lick event (Fig. 4*F*) were both significantly shorter for active trials (auditory cue response, *p* = 2.81 × 10^−74^, KS test; visual cue response, *p* = 8.84 × 10^−23^, KS test; *N* = 337 passive vs 1479 active trials). Thus, we concluded that mice learned to respond proactively in the active trials, as compared to their behavior in the passive trials. Our results indicate that mice can associate the sensory feedback to their active behavior.

**Figure 5.**
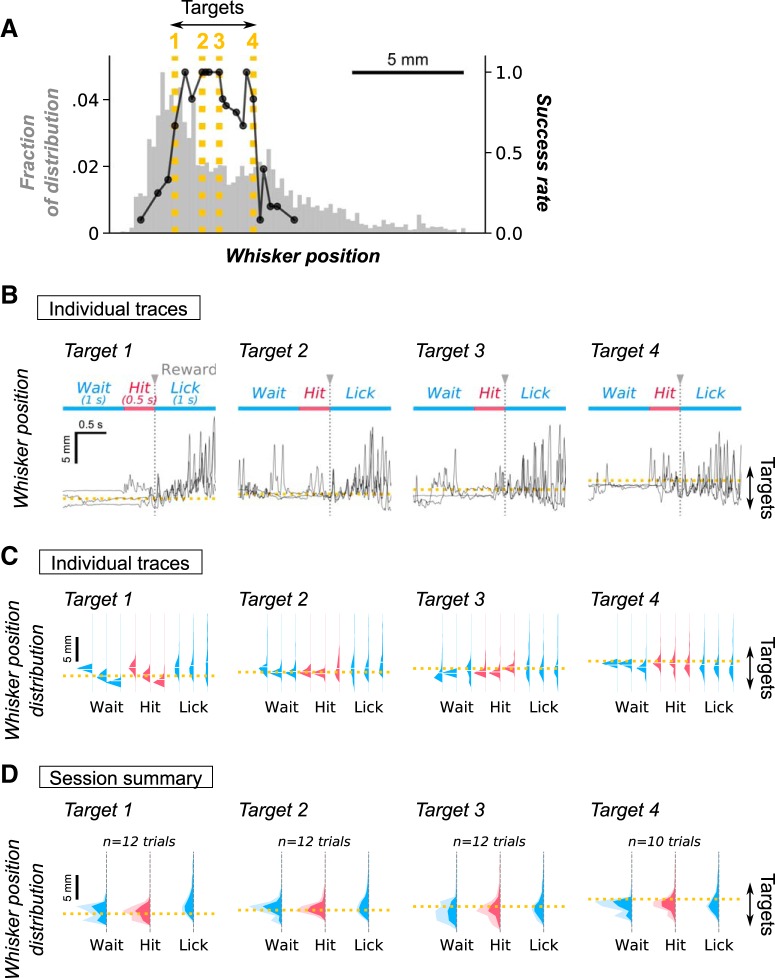
Behavioral responses to changing target positions. ***A***, Psychometric responses to changing target positions. The distribution of whisker positions in the initial 30-s period of the session is plotted as a histogram (gray). The distribution is from a period during the spontaneous whisking epoch, when there was no task. The success rates for mice positioning their whiskers in the course of the Active task, that required mice to position their whiskers to target locations, are plotted as lines (black in the foreground). Note that the virtual target regions were changed randomly after every 6–12 trials. The same target regions could appear multiple times intermittently. The *x*-axis for the black lines, the plot of success rates, corresponds to the target position. The dotted lines represent the target positions used in ***B–D***, with the number on the line indicating the corresponding target position. ***B***, The third, fourth, and sixth successful trials of whisking behavior when the target position was varied during a single session are plotted here. ***C***, Distribution of whisker positions during different behavioral phases of each trial. A histogram is generated for each representative trial shown in ***B***. The three behavioral phases include those before the animal whisks to the target (Wait), in approach to the target (Hit), and after obtaining reward (Lick). ***D***, Histograms of whisker distributions averaged across trials throughout the single session. The mode of the distributions of whisker position as the animal approached the target (Hit) correlated with the virtual target positions.

Although mice learned a behavior during the task, it was not clear that they associated the position of the whisker with any sensory feedback. To examine whether mice learned to reposition their whiskers when the virtual target location changed, we varied target positions back and forth during single sessions (Fig. 5*A*). Each target position was used until mice had made 6–12 attempts to locate the target. Plots of whisker positions during individual trials showed that mice learned to position their whiskers to a virtual target (Fig. 5*B*).

To examine the strategy that mice employed in search of the target, each trial was split *post hoc* into three phases of behavior: (1) the “Wait” epoch before mice started to move whiskers (0.5–1.5 s before reward); (2) the “Hit” epoch in which mice move their whiskers to the target area (0.5 s before reward); and (3) the “Lick” epoch (0–1 s after reward delivery), in which mice licked a lick tube to obtain a reward. As the target position moved (Fig. 5*A–D*, dotted lines), the distribution of whisker positions also moved toward the target (Fig. 5*B*). As the target locations became more difficult, i.e., required larger protractions, the starting position during the Wait phase did not shift when the target position shifted (Fig. 5*B*, right most panels). But in these trials, when the animal moved its whiskers, the position of the whisker during the Hit phase shifted toward the target position. Using these traces of whisker position, we plotted single trial histograms in the trial-based phase of behavior (Fig. 5*C*). We also plotted the average of whisker position for trials that had the same target location (Fig. 5*D*). The plots show that, especially in the Hit phase, distribution of whisker positions shifts toward the target location. Although there is a large distribution of whisker positions during each behavioral phase, i.e., mice move their whiskers back and forth through the same spatial location, our results indicate that mice adapted their behavior to target position and move their whiskers to target locations, especially during the Wait and Hit phases.

**Figure 6.**
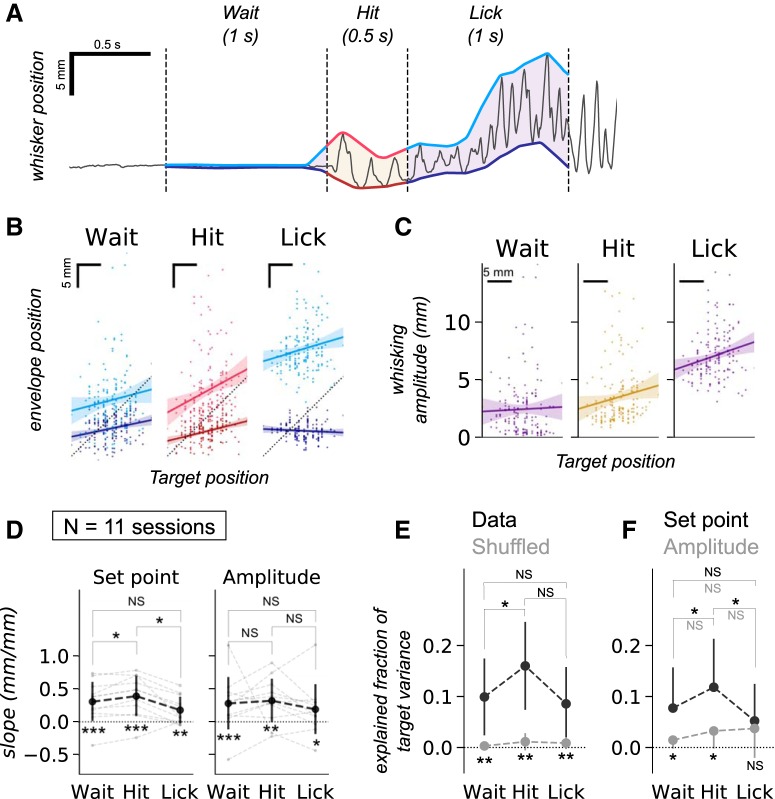
Strategy of whisker positioning with respect to changing target positions. ***A***, Computation of upper and lower bounds of whisker positions during each successful trial. Lower and upper bounds of high-speed-tracked whisker positions were computed using a 100-ms radius sliding window. ***B***, Linear regression of the upper and lower bound related to changes in target position. Each dot corresponds to a target position and is the average value of lower or upper bounds during each of the behavioral phases. There was a shift in the envelope of whisker positions as the target position moved further away. ***C***, Linear regression of whisking amplitude with respect to changing target positions. Data are from the same session as in ***B*** and are plotted similarly. Whisking amplitude was computed as the difference between lower and upper bounds. ***D***, Slopes of linear regression of set points and whisking amplitudes during different behavioral epochs and phases. Data are from 11 behavioral sessions in two animals. A Mann–Whitney *U* test was performed to examine whether the set of slopes for each trial phase were significantly larger than zero (symbols at the bottom; ***p < 0.001, **p < 0.01, *p < 0.05). Wilcoxon signed-rank test with Holm–Bonferroni correction was used to compare values between pairs of different trial phases (symbols at the top; *p < 0.05, NS, *p* > 0.05). ***E***, Multivariate linear regression analysis. The variance of the actual whisker positions (black) explained the variability of target positions more effectively than the variance of the shuffled target positions (gray; symbols at the bottom; **p < 0.01, Wilcoxon signed-rank test). The variance in Hit phase explained a significantly larger portion of the variability in target positions than during the Wait phase (symbols at the top; *p < 0.05, NS, *p* > 0.05, Wilcoxon signed-rank test with Holm–Bonferroni correction). ***F***, Comparison of regression coefficients for set points (black) and amplitudes (gray). Variability of set points were found to explain target variability more than variability of amplitudes do, during the Wait and Hit phases (symbols at the bottom; *p < 0.05, NS, *p* > 0.05, Wilcoxon signed-rank test). Variability of set points reflected target variability more during the Hit phase than during the Wait or the Lick phases (black symbols at the top; *p < 0.05, Wilcoxon signed-rank test with Holm–Bonferroni correction). There was a tendency for amplitude values to increase as the trial proceeded from the Wait through the Hit to the Lick phases, but there was no significant difference between them (gray symbols at the top; NS, *p* > 0.05, Wilcoxon signed-rank test with Holm–Bonferroni correction). Refer to the main text for the exact *R* ^2^ and *p* values.

To assess whether mice change the set point of whisker position, i.e., hold their whiskers at or near the target location, or make large amplitude movements of their whiskers, as target positions change, we computed upper and lower bounds (an envelope) of whisker motion during each trial (Fig. 6*A*). The lower bound of the envelope was defined as the set point of the C1 whisker and the difference between the upper and lower bound of the envelopes was defined as the whisking amplitude. The analysis revealed that the set point position and target position were correlated with each other during the Wait and Hit phases of the task, whereas this tendency was less obvious during the Lick phase (Fig. 6*B*). The whisking amplitude also tended to increase as the target moved further away from the resting set point of the whiskers (Fig. 6*C*).

To examine how the position of the whiskers changed with the different target locations, we quantified the slope of whisker position for 11 different behavioral sessions from two animals. The set point of whisking was significantly correlated with the target position and was significantly more correlated with target during the Hit phase than in the Wait and Lick phases (Fig. 6*D*, left). There was no significant effect of changing the target location between the Wait, Hit, and Lick phases on the amplitude of whisking (Fig. 6*D*, right).

Finally, to examine the individual contributions of the set point and whisking amplitude during the 3 phases of behavior, we used a multivariate regression model (for a detailed description of the model, see Materials and Methods). The analysis revealed that for every trial, and for each phase of the task, the variability in set points and amplitudes explained some of the variance in the position of the target. The variance explained by whisking parameters was significantly reduced when target position data were independently shuffled (Fig. 6*E*). The *R* ^2^ value for Wait phase was 0.100 ± 0.079; Hit phase was 0.160 ± 0.090; and Lick phase was 0.086 ± 0.076, whereas the *R* ^2^ values for target-shuffled dataset were: Wait, 0.003 ± 0.003; Hit, 0.011 ± 0.018; and Lick, 0.009 ± 0.012. These results suggest that, while the correlations are not high, possibly because each session has multiple and changing target locations, because mice were not yet expert in the task, or because mice change their whisking strategy as the target location changes, mice do indeed learn to position their whiskers to the target position.

There was a trial phase dependent component to these effects. The *R* ^2^ values were significantly larger for the hit phase than for the Wait phase (*p = 0.038, Wilcoxon signed-rank test with Holm–Bonferroni correction), implying that the whisker movements during the Hit phase reflected the variability of target positions more than the whisker positions during the Wait phase. But there were no significant differences between the Hit and Lick phases (*p* = 0.082) or the Wait and Lick phases (*p* = 0.182).

To examine the behavioral strategy of the mice in greater detail, we computed the fraction of target position variance explained by variance of set points and variance in whisking amplitude. The shuffled set-point position *R* ^2^ value was subtracted from the actual *R* ^2^ value, to generate the fraction of target position variance explained by set points during the Wait phase (0.077 ± 0.080), the Hit phase (0.118 ± 0.095), and the Lick phase (0.052 ± 0.073). Following a similar manipulation for the whisking amplitude, the fraction of target position variance explained by variance in the whisking amplitude was 0.015 ± 0.017 in the Wait phase, 0.033 ± 0.035 in the Hit phase, and 0.037 ± 0.058 in the Lick phase.

In both the Wait and the Hit phases the set points explained more of the target position variability, than the whisking amplitude did (Fig. 6*F*, black vs gray plots; Wait, *p = 0.016; Hit, *p = 0.041, Wilcoxon signed-rank test). There was no significant difference in the variance explained by the two parameters for the Lick phase (*p* = 0.182).

Additionally, the set point during the Hit phase explained a significantly greater portion of the variance than it did in the Wait or Lick phases (Fig. 6*F*, black plots, Hit vs Wait, *p = 0.033; Hit vs Lick, *p = 0.013, Wilcoxon signed-rank test with Holm–Bonferroni correction). This observation was consistent with the plot of the whisker position slopes in the different trial phases (Fig. 6*D*, left). There was no similar significant effect for the amplitude of whisking (Fig. 6*F*, gray plots; Wait vs Hit, *p* = 0.547; Hit vs Lick, *p* = 0.722; Wait vs Lick, *p* = 0.572, Wilcoxon signed-rank test with Holm–Bonferroni correction). Taken together, these results indicate that mice learn to position their whiskers to the target, and they primarily adjust the set-points in the Wait and the Hit phases.

## Discussion

Here, we have established real-time tracking of whisker position that can be used to generate feedback at low latency, i.e., within 2 ms. In doing so, we have created a virtual feedback environment, one in which the movement of whiskers to particular locations in space elicits reward. Mice respond to changes in the invisible target-locations by changing positions of their whiskers. In principle, whisker movement or movement of any part of the body, to any and all points in space around the animal, can be used to rotate real or virtual platforms and to deliver optogenetic stimuli to the brain.

### Comparison to existing real-time approaches

Creating a virtual feedback space around the animal requires fast and flexible behavioral tracking and feedback from the movement sequences. One state of the art strategy for tracking behavior is to use DeepLabCut (Mathis et al., 2018). With some training, the algorithm can be used to track the movement of any part of the body, but to date the real-time latency for tracking behavior with DeepLabCut ranges around 90 ms, including acquisition latency (Forys et al., 2018), or 50 ms without accounting for the time necessary for acquiring a video frame (Štih et al., 2019). Another state-of-the-art approach is to use FPGAs to compute object positions frame-by-frame, but these methods are computationally costly and so far have not been deployed in real time (Guo et al., 2015).

A common alternative to the marker-less approaches is to use markers. When markers are applied to the body, individual parts of body can be tracked with both temporal and spatial precision. Markers have been used in a variety of settings including in cinematics, sports medicine, and robotics (Marey, 1883; Winter et al., 1972; Jarret et al., 1976) and have been deployed in real-time applications (Michael et al., 2010). Motion tracking with markers consists of affixing a light-reflective or light-emitting spherical marker on a part of the body and estimating the location of the marker in 3D, at submillimeter spatial accuracy (Cappozzo et al., 2005). Spatial and temporal estimation based on conventional or depth-sensing videography can be rapid, with fast tracking speeds and latencies as short as 1.5 ms (Muro-de-la-Herran et al., 2014). Alternatively, when wearable sensors are used as active markers, e.g., accelerometers and magnetic sensors, they can have an update rate of >1 kHz (Sabatini, 2011).

These marker-based methods have also been used in animals, including mice (Bélanger et al., 1996; Leblond et al., 2003; Abbas and Masip Rodo, 2019). Markers have been used to track mouse whiskers off-line in 3D using multiple high-speed cameras (Snigdha et al., 2011), or to track mouse reaching behavior in real time at 10-ms latency (Becker and Person, 2018). Because reflective markers are often too large to be affixed onto multiple body parts of small animals, methods that employ painting or dying have also been developed for off-line tracking of rat whisking (Rigosa et al., 2017) or real-time tracking of fruit fly locomotion at 80 Hz (Kain et al., 2013). One inexpensive, easy to use paint-based real-time method is the use of color tracking with a cheap Pixy camera that works at a temporal resolution of ∼30 ms (Nashaat et al., 2017).

Yet another approach for fast feedback is to use CCD arrays (Bermejo et al., 1998) or photodiodes receiving infrared laser beam positioned at single predetermined points in space (O’Connor et al., 2013) for tracking movement. These sensors detect events almost as rapidly as they occur, but the approaches are less flexible than those described above. In addition, in the case of CCD arrays especially for the slenderer mouse whiskers, a light weight marker has to be affixed to whiskers to enhance their detectability.

In contrast to these other approaches, the neuromorphic camera-based FastEvent system achieves fidelity of tracking, comparable to that achieved with offline, high-speed camera-based tracking, and it generates feedback rapidly within 2 ms. Currently, we use inexpensive Arduino systems to control the input and output, but the feed-back time may be reduced even further by changing the input output control of the driver boards. Another important advantage of the FastEvent system is its capability of online configuration. The graphical functionality of the jAER code makes it possible to set and reset the ROI and the target region online while the animal is performing the task.

### Algorithmic considerations

Our FastEvent system achieved the real-time low-latency feedback principally because of the following two factors. The first factor is the design of the DVS neuromorphic camera. Its sensor units work on the principle of the retina as luminance change detectors, and it can transmit information about motion in the field of view in a compressed, frameless representation. The use of event-based neuromorphic cameras such as DVS cameras thus helps increase the transmission and computation efficiency. The second reason for the low latency is based on simple assumptions that we make in tracking (1) there is only one object to track in the field of view, and that (2) the tracked object appears consistently in the field of view, even if its shape and size change in the course of imaging. These assumptions led us to implement a center-of-mass approach.

While our algorithm is simple enough to run within tens of microseconds on a standard PC, it is not suitable for tracking the pose of an animal, or tracking the movement of multiple body parts simultaneously (Mathis et al., 2018), or tracking the curvature of a whisker at a point of contact (Huet et al., 2015). Some of these limitations of our system can potentially be overcome by implementing additional more complex real-time computations. Many machine vision studies using DVS cameras report that it is possible to rapidly extract different image features from the field of view (Conradt et al., 2009; Grompone von Gioi et al., 2012; Lagorce et al., 2015; Vasco et al., 2016; Mueggler et al., 2017; Everding and Conradt, 2018). But as the complexity of computations increases, the time taken to track in real-time increases, and it may therefore become necessary to pre-compute the computationally expensive calculations (Lagorce et al., 2015).

Note additionally, that in our experiments with the plucked whisker we achieved submillimeter accuracy. But in experiments with awake animals, our accuracy for locating a whisker was on the order of a millimeter. One possible explanation for this difference in performance is that in the awake behaving animal, there is interference, there is movement of other, from the non-labeled whiskers. This type of contamination can be limited by (1) removing the source of potential contamination, e.g., by trimming other whiskers, (2) enhancing the luminance of the object of interest, e.g., by using the UV paint (Nashaat et al., 2017), as well as (3) selecting the ROI to track objects. Alternatively, imposing some spatial constraints on the update of tracked positions in MeanTracker could be used to distinguish the object of interest from the irrelevant objects.

The image-processing approach for providing a feedback based on object positions may at first seem to be an overkill, compared with more conventional approaches such as the use of photodiodes (Bermejo et al., 1996, 1998; O’Connor et al., 2013). But the FastEvent system provides the experimenter with more freedom and flexibility in the experimental design. For example, with this approach, it is possible to reset the ROI for object tracking, while also resetting the virtual target regions during a behavioral session. This feature allows more variation in the experimental design, i.e., precise rules for rewarding mice or for optogenetic stimulation. The pixel array-based design of the neuromorphic camera makes it possible to generate a dense map of the animal’s behavioral strategy by changing the virtual target position pixel by pixel while monitoring the neural and behavioral activity. Lastly, the position of the virtual target can be controlled dynamically in the course of an experiment, in accordance with the animal’s behavioral state. The FastEvent system thus provides an efficient method for fine-tuning the experimental design easily, rapidly, and flexibly.

Real-time tracking approaches like the one we have described here can be used to generate patterned stimulation of the brain, or to move real and virtual environments around animals. We expect this tool set to be deployed and used to study the rapid reconfiguring of activity in neural circuits as animals adapt to a dynamically changing environment.

## Synthesis

Reviewing Editor: Liset Menendez de la Prida, Instituto Cajal CSIC Decisions are customarily a result of the Reviewing Editor and the peer reviewers coming together and discussing their recommendations until a consensus is reached. When revisions are invited, a fact-based synthesis statement explaining their decision and outlining what is needed to prepare a revision will be listed below. The following reviewer(s) agreed to reveal their identity: Mackenzie Mathis, Jason Ritt.

Your paper was evaluated by two independent reviewers and myself. We all agree that while the approach is not entirely novel, there is value in introducing a novel camera-based system for closed-loop applications in neuroscience. However, reviewers indentified some issues that require further consideration and substantial revision. We feel you should be able to address these comments in less than two months. Therefore our recommendation is to revise and re-review.

Below a synthesis of the comments:

1- A main critique is that the terminology may be incorrect. In computer vision “real-time” means an algorithm can process data at the same rate at which is is recorded (i.e. if a camera captures at 30 Hz, then if the software can offline or online process the data at >=30 Hz, it is “real time.”). What the authors show here is that they can process in **real-time + low latency (RT-LL)**, which is an important advance in neuroscience research. Thus, we suggest the authors revise the terminology and title accordingly, and also discuss work that is “low latency” in neuroscience, i.e. a lick-port is an analog way to rapidly give low-latency feedback, but image based is quite challenging, especially non-marked based.

2- Similarly, while even “state of the art” tracking of animals (the authors mention DeepLabCut as an example of this) can be run in real-time (https://www.biorxiv.org/content/10.1101/457242v1), they are so far only shown to be higher-latency (there is a pre-print on using this online here: https://www.biorxiv.org/content/10.1101/482349v1 but with considerable latency ~90 ms, and here in PlosCB with ~50 ms https://journals.plos.org/ploscompbiol/article?id=10.1371/journal.pcbi.1006699 vs. the authors impressive 2 ms reported here!). Therefore, I think they should instead say that while real time can be achieved with deep learning, low latency has yet to come to fruition. However, since here the authors do use markers on the whiskers, they need to consider marker-based tracking too. Here, there are other reports in the literature of low-latency visual-based tracking with markers, such as this paper: https://www.biorxiv.org/content/biorxiv/early/2018/05/25/330555.full.pdf

“ We first adapted a motion capture system designed for human kinematic analysis to the scale of mouse reach behavior in order to track the 3D position of a mouse's paw. Our kinematic tracking system had a spatial resolution of 150 μm and

tracking latency of less than **1 ms**”

Therefore, this body of work (i.e. marker-based tracking) should be mentioned. Author(s) may wish to discuss their work in the broader context of low-latency and DVS tracking (here is one such example: https://www.ncbi.nlm.nih.gov/pmc/articles/PMC5825909/) and marker-based tracking (i.e. example above).

3- We feel the ms suffers from insufficient technology background. The DVS and jAER will not be familiar to the eNeuro audience, and the existing references are limited and obscure, so much more explanation and background on these technologies should be provided in this paper.

As a specific example, a core component of the paper is feeding the TTL pulse back in to the DVS to measure latency, but it is unclear exactly what this means (there is an auxiliary TTL input? with what polling time constant? the LED is captured by the visual sensor?).

4- The ms also fails to properly quantify system performance. The presented technology is a tracking system used for real time feedback, and their primary claim of quality is a latency of 2 ms, but the analysis of tracking quality (Fig. 2) is performed in the frequency domain. This is, frankly, mystifying. For behavioral trials lasting a second, and reward events lasting only a fraction of a second, comparing time series over a full minute (2A) and performing frequency domain analysis is nearly meaningless. The wavelet spectrograms do not convey obviously useful information. If the authors are imagining a type of experiment where feedback is conditioned on something like whisking rate, then millisecond scale feedback is no longer essential (20 Hz corresponds to a time scale of 50 ms, and epochs of whisking occur over hundreds to thousands of milliseconds). Moreover, many existing computational systems can already handle such frequency domain criteria. Given the absence of connection to the behavioral training, it is unclear that any wavelet analysis is needed at all.

Instead, a quantification of performance including max and average/RMS deviations (from high speed video offline tracking) would be much more interpretable for most readers. The authors should report traditional quality metrics, along the lines of squared error averaged over time, and, better, some measure of rate or fraction of false triggers (where the DVS reported a position that lit the LED that was not consistent with the high-speed video reconstruction). Indeed, the traces in 2A reveal several large jumps in the DVS track without a corresponding change in high speed video. Such instantaneous jumps in a thresholding real time feedback system can present major challenges in behavioral training, for example by generating intolerably many false positives. How often do they occur? What fraction of trials might be disrupted by these events? It is impossible to tell from Fig. 2, but there may also be false negatives, and these should then be included in analysis as well.

Also fundamental, Fig 3 shows the latency from the program detecting an event to producing a pulse back at the camera surface. It does not measure the latency from the event itself. Practically all tracking systems have a short latency from the event detection to the output. It's the tracking latency from the actual event (whisker motion) that matters. In this case the authors may be underreporting their response latency (though I suspect by only a small amount, if so).

The core issue is that most systems have fundamental speed-accuracy tradeoffs. If you make the system more sensitive to motion (for example, by lowering the amount of weighted averaging), you often generate more false triggers due to noise. If some kind of smoothing is required to suppress errant triggering, then that may impact things like the reported 2 ms latency. The manuscript does not analyze the system's performance in a way that would help readers understand the necessity or impact of these tradeoffs for this system. They mention setting the time constant in an ad hoc manner, but it would be more informative for them to quantify the range of choices available to potential users, for example by assessing tracking quality as they sweep that parameter.

For all of these system characterization steps, the authors could use mechanically driven plucked whiskers or robots to better quantify the precision of tracking and the latency from motion to stimulation. The behavioral proof-of-concept is interesting and important, but the details should be both easier to measure and more precise using mechanical devices with known ground truth.

5- Issues with behavioral analysis: Fig 4E: This presentation of results is a little odd. First, performing individual comparisons at every time point is dubious statistics; it is doubtful from this presentation that the significance markers around time zero correspond to anything other than noise. Second, the region in which the Active curve does seem to lie reliably above the Passive curve occurs more than a second before the reward time, which seems to be largely irrelevant to the task (from C and D, the meaningful part of the trial seems to take about 250 ms prereward, consistent with common mouse behaviors). Perhaps there is a significant rise of the Active above the Passive just before time zero. If so, that is where the analysis and figure should be focussed, and possibly using more robust quantifications (such as looking for temporal shifts in cumulative event functions). But more fundamentally, why is the rate of “whisk events” (which I think may be misnamed; these are threshold crossings, without any effort to parse the whisker motions into identifiable discrete whisks) a good measure in the first place? Doesn't the animal need only a single crossing to enable reward?

Fig 5D: Again, the multiple comparisons of individual tests at each distance seems inappropriate. I think what the authors intend is closer to a KS test. More importantly, why would the Wait and Hit distributions be similar?

Line 269: “To address this, we varied target positions back and forth during single sessions, from the point behind the position where mouse normally locates the C1 whisker, to more distant point that the mouse cannot reach without whisking (Figure 5A)”. Using the scale bar, the target positions in 5A appear to vary by about 2.5 mm, which is a very small change. How does that variation square with this claim?

More generally, it is difficult to tell how much the mice are adjusting their setpoints and “searching” for the threshold, instead of whisking more vigorously. The small range of target positions falls entirely within “normal” whisking amplitudes. Since the task requires only threshold crossing it's not clear why the mice would do more work, anyway. Whisker position histograms are not as useful for this question, since they confound setpoint, whisk amplitude, and whisk frequency. In the examples shown, some of the highest setpoints are in the first panel, where the target is the most posterior, and in the session averages (5C) the histograms look fairly similar at all target positions. To show “mice learned to position their whiskers to a virtual target” (274) it seems necessary to distinguish setpoints from whisks (rather than collapse them all into histograms), or (better but harder) alter the task in some way that requires the animals not just to cross, but to find the target (as, for example, in work by Ziegler, or examples from Kleinfeld or Carmena).

The arguments become more muddled as this section continues. Even granting the bootstrap “control” (which is incompletely explained), why does a deviation in Wait and Hit histograms (relative to Lick) show the animals learned a target position, as opposed to showing they whisk differently in different trial epochs? Why doesn't 5D continue the analysis of true whisker position separated by target position, rather than implicitly and somewhat confusingly embedding that target inside a deviation?

6- Insufficient discussion on previous work and current limitations: Although the authors do correctly address some limitations of their system in the Discussion, the limitations appear more substantial than the writing would suggest. Most important is the inability to track multiple objects or spatially extended structures. They compare their system to that of Matthis and colleagues, which by design performs pose/joint estimation, not simple location tracking. Further, whisker curvature is widely considered an important variable in rodent touch, and it is unclear how the authors would extend DVS to work with curvature (without adding computational stages that would substantially damage latency). The paper would be improved if the authors discussed in some detail how their system might address these more challenging problems, at least in principle, even if not immediately demonstrated in behaving animals.

Consistent with the sparse discussion of the system's limitations is missing background on other real time feedback training in the whisker system. Work from Ziegler and colleagues, that trained rats to hold specific whisker positions using linear CCD arrays, is now considered classic, and should be referenced. The introduction and discussion should also reference work very similar to the current paper, by Venkatraman and Carmena, and by O'Connor, et al. Specifically, it appears everything done by the authors in this paper could have been (and to some degree, was) done in those earlier works --- at the simplest extreme, using photodiodes. Programmability and flexibility are worthy properties, but were not compellingly demonstrated in the current paper with such a simple task. At least some comparison with these earlier efforts, and ideally some demonstration that hints at broader applications for the DVS approach, would improve the paper.

Although the real time feedback is different, the Sofroniew, et al studies on wall following should also be referenced.

7- Lack of clarity in methods: In general the Methods are presented in a convoluted and confusing order. An early overview paragraph like “We trained head fixed animals to detect auditory and visual cues...”, before the detailed methods, could significantly help orient readers.

Surgery is described before it's stated what will be implanted or why in terms of behavior; a head post is mentioned, but so is black dental acrylic, which left me wondering why there was no optical fiber, especially as optogenetics is mentioned in the Abstract, but the paper does not actually use optogenetics.

The behavioral description jumps forward and backwards between what trained animals ought to do and what it takes to get animals trained. It's reasonable to present task structure before training for complex tasks, but in this case it seems like it would be simpler and clearer to describe the behavioral methods in experiment chronological order (i.e. start with habituation to head fixation, then shaping, then Active/Passive...). As written the Methods section takes too much work to figure out, for what is ultimately a very simple behavior.

It is especially important to make clear that this is a visual feedback task, not a neurofeedback task. While from a hardware perspective it might not make much difference, the current presentation borders on disingenuous in implying that the authors have already demonstrated optogenetic feedback using their system. I suggest clarifying the Abstract and Introduction on this point, as well as Methods.

Line 139: It's not immediately clear what the ij indices are indexing. I gather i indexes discrete (update or “packet”) time, as distinct from true, continuous time t, and j indexes a set of simultaneous events received at the ith update. But one might wonder what happens if there are multiple objects, and j is actually indexing those. Instead, the authors made sure there was only a single high luminance object in view, and then averaged over the pixels covering that object (a “center of mass”). The language here could be clearer.

The time constant of 200-300 microseconds is presumably included to smooth over electronic issues, and not whisker trajectories. It would be helpful to have more information about the statistics of packet arrivals (how often is there data with which to update x?), and specifically if/how packet updates change with motion in the frame. How bad is the “update noise”?

Other comments:

The authors should clarify a technical detail in the behavior: did the animals have to hold their whiskers in the LED On location to begin licking for reward, or did any “instantaneous” hit of the on location enable licking/reward for the rest of the trial?

Lines 81-83: “Animals were trained to lick the drop of water (2-5 μL) after the auditory Go-cue turned on, only after a second cue, a LED light was also active during this period.” Fix grammar, and clarify the paradigm (is the LED the “second cue”, or another tone?). Alternatively, just cut these lines as they seem to be redundant with the next paragraph.

Line 86: Is the Suppressed period cued to the animal? Waiting, Suppressed, Cued and Post-Response periods described in Methods do not line up as clearly as they should with the Wait, Hit, and Lick periods in the figures; some tightening of language would clarify the task structure.

Line 124: The video acquisition could be clarified. “each video was triggered, and 2.5 seconds of video before reward delivery”. How was triggering fixed-length videos performed in the active phase, when the timing varied with animal behavior?

Line 152: “the XYFilter for restricting the ROI”. What was the ROI, both in terms of pixels, and in area/diameter at the place of the whisker?

Line 167: “Whisking motion and licking as well as the triggers for cues, trials and rewards were monitored and acquired with Spike2 software, via a 1401 data acquisition interface (Cambridge Electronic Design, UK).” More detail should be given here. What lick sensors were used, with what properties (e.g. latency)? And what does “whisking motion” mean for the behavioral subsystem, given that the point of this paper is to introduce and describe a high performance whisking sensor?

Fig 3C: there is no scale on the vertical axis, and it is not even explicit that the baseline counts are actually at zero.

Fig 4A: “lick spout...was positioned for easily monitoring the animal's lick behavior.” How was the monitoring actually performed?

Fig 5A: The caption is a bit confusing. The authors should state explicitly that each panel corresponds to a different target location, and shows three trials (selected how?).

Line 373: “dark light”. Do they mean dark field illumination?

There are many missing details. What kind of camera lenses? What kind of illumination (what devices, what wavelength, what spatial spread, what orientation relative to the cameras, how bright)? What are the camera and DVS native ROIs (in pixels and mm)? What is the restricted ROI used in experiments (also, what the authors are calling an ROI may be better described as a “target box”)? What kind of computer, what kind of OS, what kind of interface between camera/DVS and computer, what kind of interface between the computer and the Arduino (these and similar details all relate to the claimed 2 msec latency)?

Fig 1B: I can't tell how much the DVS actually relies on the paint; it seems to catch a wider segment of the whisker, possibly dependent more on the direction of the illuminator. Also, there seems to be some cropping in DVS at 44.2 ms (on the left side), that doesn't match the horizontal extent at 0 ms; is the black background here the actual ROI that led to triggering, or something else? Also, since we aren't given the relative orientations of the camera and DVS, it's hard to know how similar the two representations should be in the first place.

line 49 GPU is misspelled (GPGPU)

The software used should be cited not just mentioned in the methods: i.e pandas, Python, etc. Please find the citations here: https://www.scipy.org/citing.html

## Author Response

Dear Dr. Menedez de laPrida,

In line with the reviewer's suggestions, comments and criticism, we have revised our manuscript. We have performed new experiments, used plucked whiskers, tested whether 2 ms latency is the total latency, analyzed the behavioral outcome in multiple ways, and have given more detailed background about the DVS neuromorphic camera system. We look forward to hearing from you.

-

Responses to reviewer comments

Major comments

Comment #1

A main critique is that the terminology may be incorrect. In computer vision “real-time” means an algorithm can process data at the same rate at which is is recorded (i.e. if a camera captures at 30 Hz, then if the software can offline or online process the data at >=30 Hz, it is “real time.”). What the authors show here is that they can process in **real-time + low latency (RT-LL)**, which is an important advance in neuroscience research. Thus, we suggest the authors revise the terminology and title accordingly, and also discuss work that is “low latency” in neuroscience, i.e. a lick-port is an analog way to rapidly give low-latency feedback, but image based is quite challenging, especially non-marked based.

Following on the reviewers' suggestion, we have added to our expression and are using “real-time” and combining this with “low-latency”, where applicable, throughout the manuscript (lines 8, 18, 67, 75, 510, 618, 849).

Comment #2

Similarly, while even “state of the art” tracking of animals (the authors mention DeepLabCut as an example of this) can be run in real-time (https://www.biorxiv.org/content/10.1101/457242v1), they are so far only shown to be higher-latency (there is a pre-print on using this online here: https://www.biorxiv.org/content/10.1101/482349v1 but with considerable latency ~90 ms, and here in PlosCB with ~50 ms https://journals.plos.org/ploscompbiol/article?id=10.1371/journal.pcbi.1006699 vs. the authors impressive 2 ms reported here!). Therefore, I think they should instead say that while real time can be achieved with deep learning, low latency has yet to come to fruition. However, since here the authors do use markers on the whiskers, they need to consider marker-based tracking too. Here, there are other reports in the literature of low-latency visual-based tracking with markers, such as this paper: https://www.biorxiv.org/content/biorxiv/early/2018/05/25/330555.full.pdf

“We first adapted a motion capture system designed for human kinematic analysis to the scale of mouse reach behavior in order to track the 3D position of a mouse's paw. Our kinematic tracking system had a spatial resolution of 150 μm and tracking latency of less than **1 ms**”

Therefore, this body of work (i.e. marker-based tracking) should be mentioned. Author(s) may wish to discuss their work in the broader context of low-latency and DVS tracking (here is one such example: https://www.ncbi.nlm.nih.gov/pmc/articles/PMC5825909/) and marker-based tracking (i.e. example above).

We have added a section on behavioral tracking methods in the introduction (lines 46-60) and discussion (lines 629-632, 636-646). We also added a section on event-based tracking approaches (lines 69-72).

Comment #3

We feel the ms suffers from insufficient technology background. The DVS and jAER will not be familiar to the eNeuro audience, and the existing references are limited and obscure, so much more explanation and background on these technologies should be provided in this paper.

As a specific example, a core component of the paper is feeding the TTL pulse back in to the DVS to measure latency, but it is unclear exactly what this means (there is an auxiliary TTL input? with what polling time constant? the LED is captured by the visual sensor?).

We have added more background description on the DVS camera and jAER, at the beginning of the Materials and Methods section within the context of our work (lines 79-122). We have also added details about how the latency is measured, and the TTL is recorded in the methods section (lines 168-176, 385-396).

Comment #4

The ms also fails to properly quantify system performance. The presented technology is a tracking system used for real time feedback, and their primary claim of quality is a latency of 2 ms, but the analysis of tracking quality (Fig. 2) is performed in the frequency domain. This is, frankly, mystifying. For behavioral trials lasting a second, and reward events lasting only a fraction of a second, comparing time series over a full minute (2A) and performing frequency domain analysis is nearly meaningless. The wavelet spectrograms do not convey obviously useful information. If the authors are imagining a type of experiment where feedback is conditioned on something like whisking rate, then millisecond scale feedback is no longer essential (20 Hz corresponds to a time scale of 50 ms, and epochs of whisking occur over hundreds to thousands of milliseconds). Moreover, many existing computational systems can already handle such frequency domain criteria. Given the absence of connection to the behavioral training, it is unclear that any wavelet analysis is needed at all.

We are bit puzzled by this comment. The goal of the original figure 2 was to compare DVS system to post-hoc video analysis in both analogue waveform and frequency domain. DVS system clearly is adequate in tracking and this can be seen in both waveform, and in the frequency domain where phase and frequency can be compared for the tracking methods.

With regard to whether 2 ms latency is ever necessary or is simply overkill, we are mystified. Considering Nyquist limits, to adequately track an object moving at 25 Hz, we would need many more points than the bare minimum to calculate a correct trajectory of the whisker. Consider the fact that when we record action potentials -- maximum firing rate is 1KHz, an adequate sampling rate is never 2KHz, it is closer to 20Khz or 40 Khz. The faster the system works, the more accurately it tracks the element. An obvious alternative method to the use of DVS or imaging methodology for tracking movement is to use EMG where tracking can work at 1KHz with little problem. But EMG is invasive, and EMG cannot track an single whisker, only the whisker pad.

We have edited this text and have added a new figure with plucked B1 whisker as Figure 2A-D.

Instead, a quantification of performance including **max and average/RMS deviations (from high speed video offline tracking)** would be much more interpretable for most readers. The authors should report traditional quality metrics, along the lines of squared error averaged over time, and, better, some measure of rate or fraction of false triggers (where the DVS reported a position that lit the LED that was not consistent with the high-speed video reconstruction). Indeed, the traces in 2A reveal several large jumps in the DVS track without a corresponding change in high speed video. Such instantaneous jumps in a thresholding real time feedback system can present major challenges in behavioral training, for example by generating intolerably many false positives. How often do they occur? What fraction of trials might be disrupted by these events? It is impossible to tell from Fig. 2, but there may also be false negatives, and these should then be included in analysis as well.

We have added a discussion point about these large jumps in the DVS tracking. These jumps arise from the adjacent unlabeled whisker moving in the region of interest. In the current experiments, the neighboring whiskers were long enough to move through the same spatial location as the labelled whisker, in the region of interest. This error is negligible if all whiskers except for the tracked one are trimmed off. The plucked whisker experiments, Figure 2A-D show the results with the trimmed whisker.

We have re-analyzed our data using the RMS error metric, and show the bounds in the histogram (Figure 2G, H). The RMS error in position was 0.6 mm (Fig 2G) and ~20 degree error in phase.

Also fundamental, Fig 3 shows the latency from the program detecting an event to producing a pulse back at the camera surface. It does not measure the latency from the event itself. Practically all tracking systems have a short latency from the event detection to the output. It's the tracking latency from the actual event (whisker motion) that matters. In this case the authors may be underreporting their response latency (though I suspect by only a small amount, if so).

To address the reviewers' concern, we added another experiment based on a pair of LEDs to measure the real latency of our system (new Figure 3D, E). The additional time added by tracking whisker motion is miniscule, about 0.1 ms. We describe the new experiment in the methods on lines 229-240 and the results on lines 497-511.

The core issue is that most systems have fundamental speed-accuracy tradeoffs. If you make the system more sensitive to motion (for example, by lowering the amount of weighted averaging), you often generate more false triggers due to noise. If some kind of smoothing is required to suppress errant triggering, then that may impact things like the reported 2 ms latency. The manuscript does not analyze the system's performance in a way that would help readers understand the necessity or impact of these tradeoffs for this system. They mention setting the time constant in an ad hoc manner, but it would be more informative for them to quantify the range of choices available to potential users, for example by assessing tracking quality as they sweep that parameter.

Following the suggestions by the reviewers, we used 3 different time-constant parameters, and estimated the effect of these time-constants and the errors that result from the different values. The integration time constant was introduced to reduce the errors introduced by noise. Increasing the time constant to 1ms, reduces the tracking efficiency, especially at higher frequencies. The results are described in the text (lines 451-472) and as a figure (Figure 2A-D).

For all of these system characterization steps, the authors could use mechanically driven plucked whiskers or robots to better quantify the precision of tracking and the latency from motion to stimulation. The behavioral proof-of-concept is interesting and important, but the details should be both easier to measure and more precise using mechanical devices with known ground truth.

As noted above, we have added an experiment with plucked whiskers. The results are described above and in Figure 2 (A-D). The reviewer suggested the possibility of using mechanical, artificial setup for profiling of the system. While we profile the system using the plucked whisker, we did not move the whisker at 20 Hz, primarily because the movement of the artificial whisker could not be controlled with sub-millimeter accuracy at high frequencies.

Comment #5

Issues with behavioral analysis: Fig 4E: This presentation of results is a little odd. First, performing individual comparisons at every time point is dubious statistics; it is doubtful from this presentation that the significance markers around time zero correspond to anything other than noise. Second, the region in which the Active curve does seem to lie reliably above the Passive curve occurs more than a second before the reward time, which seems to be largely irrelevant to the task (from C and D, the meaningful part of the trial seems to take about 250 ms prereward, consistent with common mouse behaviors). Perhaps there is a significant rise of the Active above the Passive just before time zero. If so, that is where the analysis and figure should be focussed, and possibly using more robust quantifications (such as looking for temporal shifts in cumulative event functions). But more fundamentally, why is the rate of “whisk events” (which I think may be misnamed; these are threshold crossings, without any effort to parse the whisker motions into identifiable discrete whisks) a good measure in the first place? Doesn't the animal need only a single crossing to enable reward?

We agree that our points were unclear in Fig. 4. We have focused the analysis around the threshold crossing event and have changed the analysis and display to show the threshold crossing events during the auditory cue epoch. We now plot cumulative histograms comparing the event frequency, and the latency in the passive and active behavioral conditions (Figure 4E). We also plot the cumulative histogram for the latency to the visual cue in both behaviors (Figure 4F). These figures show that there were significant differences in all of these measures for the active and passive behavioral condition. The text has been re-written accordingly (lines 524-533).

Fig 5D: Again, the multiple comparisons of individual tests at each distance seems inappropriate. I think what the authors intend is closer to a KS test. More importantly, why would the Wait and Hit distributions be similar?

According to the reviewer's comments, we changed the method of analysis (refer to the response below).

Line 269: “To address this, we varied target positions back and forth during single sessions, from the point behind the position where mouse normally locates the C1 whisker, to more distant point that the mouse cannot reach without whisking (Figure 5A)”. Using the scale bar, the target positions in 5A appear to vary by about 2.5 mm, which is a very small change. How does that variation square with this claim?

More generally, it is difficult to tell how much the mice are adjusting their setpoints and “searching” for the threshold, instead of whisking more vigorously. The small range of target positions falls entirely within “normal” whisking amplitudes. Since the task requires only threshold crossing it's not clear why the mice would do more work, anyway. Whisker position histograms are not as useful for this question, since they confound setpoint, whisk amplitude, and whisk frequency. In the examples shown, some of the highest setpoints are in the first panel, where the target is the most posterior, and in the session averages (5C) the histograms look fairly similar at all target positions. To show “mice learned to position their whiskers to a virtual target” (274) it seems necessary to distinguish setpoints from whisks (rather than collapse them all into histograms), or (better but harder) alter the task in some way that requires the animals not just to cross, but to find the target (as, for example, in work by Ziegler, or examples from Kleinfeld or Carmena).

To address this issue, we have reanalyzed the whisker movement during the behavior for whisker setpoint and whisking amplitude (Figure 5, 6). We have minimized our use of “whisking” in this context (line 534-558). In addition, we have plotted the “resting” position of the C1 whisker, and the relationship of the C1 whisker position to the target position (Figure 5A). We have also analyzed these whisker and target positions in relation to the animals behavioral state, i.e. Waiting, Hit, or Lick.

The arguments become more muddled as this section continues. Even granting the bootstrap “control” (which is incompletely explained), why does a deviation in Wait and Hit histograms (relative to Lick) show the animals learned a target position, as opposed to showing they whisk differently in different trial epochs? Why doesn't 5D continue the analysis of true whisker position separated by target position, rather than implicitly and somewhat confusingly embedding that target inside a deviation?

We have removed the bootstrap control from the analysis. We have also modified our method of analysis and have removed former Figure 5D. We have added a new Figure 6. Now we use the lower and upper bounds of whisker positions during each of the three trial phases, and analyze their relationships to target locations by fitting linear regression models. The results are described as the Figure 6. The Methods (lines 397-432) and Results sections (lines 559-615) are rewritten accordingly.

Comment #6

Insufficient discussion on previous work and current limitations: Although the authors do correctly address some limitations of their system in the Discussion, the limitations appear more substantial than the writing would suggest. Most important is the inability to track multiple objects or spatially extended structures. They compare their system to that of Matthis and colleagues, which by design performs pose/joint estimation, not simple location tracking. Further, whisker curvature is widely considered an important variable in rodent touch, and it is unclear how the authors would extend DVS to work with curvature (without adding computational stages that would substantially damage latency). The paper would be improved if the authors discussed in some detail how their system might address these more challenging problems, at least in principle, even if not immediately demonstrated in behaving animals.

The criticism that our system cannot track multiple objects in realtime or the curvature of whiskers in real time is valid. We do now note in the discussion that with tweaking of our tracking algorithm, it will be possible to track multiple objects and even the curvature in real time (lines 667-677). Also note that DeepLabCut does not perform joint estimation but predicts a set of one-versus-rest probabilities.

Consistent with the sparse discussion of the system's limitations is missing background on other real time feedback training in the whisker system. Work from Ziegler and colleagues, that trained rats to hold specific whisker positions using linear CCD arrays, is now considered classic, and should be referenced. The introduction and discussion should also reference work very similar to the current paper, by Venkatraman and Carmena, and by O'Connor, et al. Specifically, it appears everything done by the authors in this paper could have been (and to some degree, was) done in those earlier works --- at the simplest extreme, using photodiodes. Programmability and flexibility are worthy properties, but were not compellingly demonstrated in the current paper with such a simple task. At least some comparison with these earlier efforts, and ideally some demonstration that hints at broader applications for the DVS approach, would improve the paper.

We have added more direct discussion of these points (lines 641-646). Note that CCD arrays of Ziegler required the use of relatively large markers for adequate tracking of thicker rat whiskers.

Although the real time feedback is different, the Sofroniew, et al studies on wall following should also be referenced.

We added the reference as suggested by the reviewers (line 33).

Comment #7

Lack of clarity in methods: In general the Methods are presented in a convoluted and confusing order. An early overview paragraph like “We trained head fixed animals to detect auditory and visual cues...”, before the detailed methods, could significantly help orient readers.

According to the reviewers' comment, we reorganized the Materials and Methods section (lines 79-432).

Surgery is described before it's stated what will be implanted or why in terms of behavior; a head post is mentioned, but so is black dental acrylic, which left me wondering why there was no optical fiber, especially as optogenetics is mentioned in the Abstract, but the paper does not actually use optogenetics.

We have rewritten the surgical description (line 249) and removed statements about optogenetics from the Abstract.

The behavioral description jumps forward and backwards between what trained animals ought to do and what it takes to get animals trained. It's reasonable to present task structure before training for complex tasks, but in this case it seems like it would be simpler and clearer to describe the behavioral methods in experiment chronological order (i.e. start with habituation to head fixation, then shaping, then Active/Passive...). As written the Methods section takes too much work to figure out, for what is ultimately a very simple behavior.

We have rewritten this section (lines 281-319).

It is especially important to make clear that this is a visual feedback task, not a neurofeedback task. While from a hardware perspective it might not make much difference, the current presentation borders on disingenuous in implying that the authors have already demonstrated optogenetic feedback using their system. I suggest clarifying the Abstract and Introduction on this point, as well as Methods.

We have removed the mention on optogenetics from the Abstract and have left it in the Discussion (line 623).

Line 139: It's not immediately clear what the ij indices are indexing. I gather i indexes discrete (update or “packet”) time, as distinct from true, continuous time t, and j indexes a set of simultaneous events received at the ith update. But one might wonder what happens if there are multiple objects, and j is actually indexing those. Instead, the authors made sure there was only a single high luminance object in view, and then averaged over the pixels covering that object (a “center of mass”). The language here could be clearer.

We added a text to clarify our tracking method (lines 125-129).

The time constant of 200-300 microseconds is presumably included to smooth over electronic issues, and not whisker trajectories. It would be helpful to have more information about the statistics of packet arrivals (how often is there data with which to update x?), and specifically if/how packet updates change with motion in the frame. How bad is the “update noise”?

We added the information on packet update intervals (lines 104-106), and reasoning behind adjusting the integration time constant (lines 138-143).

Minor comments

The authors should clarify a technical detail in the behavior: did the animals have to hold their whiskers in the LED On location to begin licking for reward, or did any “instantaneous” hit of the on location enable licking/reward for the rest of the trial?

We added description to clarify this point (lines 314-317).

Lines 81-83: “Animals were trained to lick the drop of water (2-5 μL) after the auditory Go-cue turned on, only after a second cue, a LED light was also active during this period.” Fix grammar, and clarify the paradigm (is the LED the “second cue”, or another tone?). Alternatively, just cut these lines as they seem to be redundant with the next paragraph.

We changed the sentences to make our points clearer (line 282-283).

Line 86: Is the Suppressed period cued to the animal? Waiting, Suppressed, Cued and Post-Response periods described in Methods do not line up as clearly as they should with the Wait, Hit, and Lick periods in the figures; some tightening of language would clarify the task structure.

Following the reviewers' suggestions, we removed the descriptions on periods from our task descriptions as they are not cued to the animal (lines 298-319). We restricted our use of trial phases within the context of analysis (lines 404-432, 541-615).

Line 152: “the XYFilter for restricting the ROI”. What was the ROI, both in terms of pixels, and in area/diameter at the place of the whisker?

We added a sentence to clarify where we put the ROI (lines 179-182).

Line 167: “Whisking motion and licking as well as the triggers for cues, trials and rewards were monitored and acquired with Spike2 software, via a 1401 data acquisition interface (Cambridge Electronic Design, UK).” More detail should be given here. What lick sensors were used, with what properties (e.g. latency)? And what does “whisking motion” mean for the behavioral subsystem, given that the point of this paper is to introduce and describe a high performance whisking sensor?

We changed the description to clarify the points (lines 328-330).

Fig 3C: there is no scale on the vertical axis, and it is not even explicit that the baseline counts are actually at zero.

We added the missing scale on Figure 3C.

Fig 4A: “lick spout...was positioned for easily monitoring the animal's lick behavior.” How was the monitoring actually performed?

We changed the expression to clarify the point on Figure 4A (line 876).

Fig 5A: The caption is a bit confusing. The authors should state explicitly that each panel corresponds to a different target location, and shows three trials (selected how?).

We added a panel (Figure 5A) to show where the selected targets are, and added the correspondences to the rest of the panels. We made a statement on which trials we selected (line 920).

Line 373: “dark light”. Do they mean dark field illumination?

We clarified with the terminology regarding the illumination (lines 260, 272, 802).

There are many missing details. What kind of camera lenses? What kind of illumination (what devices, what wavelength, what spatial spread, what orientation relative to the cameras, how bright)? What are the camera and DVS native ROIs (in pixels and mm)? What is the restricted ROI used in experiments (also, what the authors are calling an ROI may be better described as a “target box”)? What kind of computer, what kind of OS, what kind of interface between camera/DVS and computer, what kind of interface between the computer and the Arduino (these and similar details all relate to the claimed 2 msec latency)?

We added more detailed descriptions in the Materials and Method sections (lines 158-167, 177-185).

Fig 1B: I can't tell how much the DVS actually relies on the paint; it seems to catch a wider segment of the whisker, possibly dependent more on the direction of the illuminator. Also, there seems to be some cropping in DVS at 44.2 ms (on the left side), that doesn't match the horizontal extent at 0 ms; is the black background here the actual ROI that led to triggering, or something else? Also, since we aren't given the relative orientations of the camera and DVS, it's hard to know how similar the two representations should be in the first place.

We added the descriptions in the Materials and Methods section (lines 255-264).

line 49 GPU is misspelled (GPGPU).

We have removed the term as we rewrote the section (lines 52-60).

The software used should be cited not just mentioned in the methods: i.e pandas, Python, etc. Please find the citations here: https://www.scipy.org/citing.html

We cited the Python and its libraries (lines 357-363).
